# Species-Wide Variation in Shoot Nitrate Concentration, and Genetic Loci Controlling Nitrate, Phosphorus and Potassium Accumulation in *Brassica napus* L.

**DOI:** 10.3389/fpls.2018.01487

**Published:** 2018-10-16

**Authors:** Thomas D. Alcock, Lenka Havlickova, Zhesi He, Lolita Wilson, Ian Bancroft, Philip J. White, Martin R. Broadley, Neil S. Graham

**Affiliations:** ^1^Plant and Crop Sciences Division, University of Nottingham, Sutton Bonington Campus, Loughborough, United Kingdom; ^2^Department of Biology, University of York, York, United Kingdom; ^3^The James Hutton Institute, Dundee, United Kingdom; ^4^Distinguished Scientist Fellowship Program, King Saud University, Riyadh, Saudi Arabia

**Keywords:** associative transcriptomics, GWAS, nitrogen, nutrient use efficiency, suberin, casparian strip, fertilizer, diversity population

## Abstract

Large nitrogen, phosphorus and potassium fertilizer inputs are used in many crop systems. Identifying genetic loci controlling nutrient accumulation may be useful in crop breeding strategies to increase fertilizer use efficiency and reduce financial and environmental costs. Here, variation in leaf nitrate concentration across a diversity population of 383 genotypes of *Brassica napus* was characterized. Genetic loci controlling variation in leaf nitrate, phosphorus and potassium concentration were then identified through Associative Transcriptomics using single nucleotide polymorphism (SNP) markers and gene expression markers (GEMs). Leaf nitrate concentration varied over 8-fold across the diversity population. A total of 455 SNP markers were associated with leaf nitrate concentration after false-discovery-rate (FDR) correction. In linkage disequilibrium of highly associated markers are a number of known nitrate transporters and sensors, including a gene thought to mediate expression of the major nitrate transporter NRT1.1. Several genes influencing root and root-hair development co-localize with chromosomal regions associated with leaf P concentration. Orthologs of three ABC-transporters involved in suberin synthesis in roots also co-localize with association peaks for both leaf nitrate and phosphorus. Allelic variation at nearby, highly associated SNPs confers large variation in leaf nitrate and phosphorus concentration. A total of five GEMs associated with leaf K concentration after FDR correction including a GEM that corresponds to an auxin-response family protein. Candidate loci, genes and favorable alleles identified here may prove useful in marker-assisted selection strategies to improve fertilizer use efficiency in *B. napus*.

## Introduction

The plant macronutrients nitrogen (N), phosphorus (P) and potassium (K) are required in large amounts by higher plants, which typically contain approximately 1.5% N, 0.2% P, and 1% K on a dry weight (DW) basis (Hawkesford et al., 2012). Large concentrations of soil N are found in organic matter, the majority of which is in the form of peptides and proteins (Jones et al., 2002). Whilst largely unavailable to plants directly (Dechorgnat et al., 2011), there is evidence to suggest that plants can acquire organic N through root uptake of free amino acids (Näsholm et al., 2009). These can be made available through the breakdown of peptides and proteins by soil micro-organisms, and may be the dominant form of N in some high latitude ecosystems (Miller and Cramer, 2004). However, the major source of N to plants in aerobic, agricultural soils is thought to be nitrate (${\text{NO}}_{3}^{-}$), which represents < 2% of soil N (Dechorgnat et al., 2011). Similarly, although total soil P content is generally considered to be relatively high, bioavailable P is present at much lower concentrations (White and Hammond, 2008). This is largely due to the ability of P to form insoluble complexes with cations including calcium (Ca), magnesium (Mg), iron (Fe), and aluminum (Al; Hinsinger, 2001). The availability of P in soils is also thought to be declining due to soil degradation, which is estimated to have affected over half of global agricultural land, and the majority of agricultural land in Africa (Lynch, 2011). The concentration of K in soils can vary greatly depending on soil type, moisture, and chemical composition (Maathuis and Sanders, 1996). Much of the K available to crops is present in the soil solution, which represents only 0.1–0.2% of total soil K (White, 2013). Whilst further sources of K from exchangeable and non-exchangeable fractions of the soil are generally available to replenish soil solution K, typically 90–98% of total soil K remains effectively inaccessible to crop plants.

Due to the highly intensive nature of modern agriculture, bioavailable supplies of N, P, and K in arable soils can quickly become depleted (Jones et al., 2013). This can lead to the manifestation of symptoms including stunting, narrow leaves, and chlorosis under N insufficiency, inhibited shoot growth under P insufficiency, and retarded growth and shoot necrosis under K insufficiency (Hawkesford et al., 2012). In order to prevent such symptoms, huge quantities of fertilizer are applied to soils every year. In 2016, worldwide N fertilizer demand was 112 million tons, and demand is estimated to be increasing by 1.5% annually (FAO, 2017). Global demand for P and K fertilizer in the same year were around 42 and 33 million tons, with demand expected to increase by 2.2 and 2.4% per year, respectively (FAO, 2017). Fertilizer use is expensive, driving up costs to both the farmer and the consumer. It can also be harmful to the environment by contributing to greenhouse gas emissions, of which agricultural fertilizer use is the main source of atmospheric N_2_O release (Snyder et al., 2009). There is also a need to reduce excessive anthropogenic nutrient inputs to aquatic ecosystems through agricultural runoffs in order to reduce eutrophication and protect drinking water supplies (Conley et al., 2009). For example, in order to return to within Earth's critical boundary set for excessive P in waterways, it is estimated that daily food supply per capita would have to drop below one tenth of the present supply under current agricultural practices and diets (Kahiluoto et al., 2013). Thus, a significant challenge in modern agriculture is the generation of new varieties better able to uptake and utilize available nutrients, in order to improve yields, whilst reducing fertilizer inputs.

*Brassica napus* L. is a crop species of global importance, encompassing oilseed varieties, commonly known as oilseed rape or canola, as well as vegetable crop types including swede or rutabaga, and leafy types including fodder and kale (Allender and King, 2010). Among major oilseeds, production of oilseed rape is the second largest behind soybean, with an estimated production of 74.28 million tons between April 2017 and April 2018 (USDA, 2018). It is also the third largest source of vegetable oil globally behind palm and soybean, with world supply predicted to be 28.84 million tons for the same period. Of this, approximately 10 million tons is estimated to be produced by the European Union, in which oilseed rape is the primary source of vegetable oil (European Commission, 2018). Only following intensive breeding programmes to reduce concentrations of toxic erucic acid and bitter glucosinolates from seed oil over approximately the last 50 years has *B. napus* reached global importance as a source of high quality vegetable oil (Allender and King, 2010). Hence, it is likely that there is still extensive scope for improvements in nutrient use efficiency in *B. napus*. For instance, N use efficiency in oilseed rape was estimated to be less than half that of barley and winter wheat (Berry et al., 2010).

There is significant potential for such improvements to be made through conventional breeding, as reflected by the variation in nutrient concentration traits previously observed (Ding et al., 2010; Bus et al., 2014; Koprivova et al., 2014; Thomas et al., 2016). For instance, in the study of Koprivova et al. (2014), leaf nitrate and phosphate concentrations varied 83- and 5-fold respectively across a population of 99 field-grown genotypes of *B. napus*. Approximately 2-fold variation was also observed in both leaf P and K concentration traits in a larger population of *B. napus* grown in compost (Thomas et al., 2016). However, efforts to breed for increased nutrient use efficiency could also benefit from the identification of genes controlling nutrient accumulation, which would allow a more targeted approach to crop improvement. In *Arabidopsis thaliana*, the genetic control of ${\text{NO}}_{3}^{-}$, P and K uptake is relatively well understood. Two gene families known as *NRT1* and *NRT2* are largely thought to co-ordinate ${\text{NO}}_{3}^{-}$ supply (Tsay et al., 2007), four major phosphate transporter (*PHT*) gene families have been described, known as *PHT1, PHT2, PHT3*, and *PHT4* (Raghothama and Karthikeyan, 2005), and K uptake is thought to be largely co-ordinated by two high-affinity transporters, HIGH-AFFINITY K^+^ TRANSPORTER 5 (*HAK5*) and ARABIDOPSIS K^+^ TRANSPORTER 1 (*AKT1*; Hirsch et al., 1998; Pyo et al., 2010). Despite being closely related to *A. thaliana*, an understanding of the genetic basis of ${\text{NO}}_{3}^{-}$, P and K accumulation in *B. napus* specifically is limited (Bouchet et al., 2016). Whilst work to identify genetic loci controlling anion homeostasis (Koprivova et al., 2014) and shoot mineral concentrations (Bus et al., 2014) in *B. napus* has been performed previously, further work is required to pinpoint specific genes and pathways that have the largest influence on such traits.

A genome-wide association study (GWAS) performed with leaf anion concentration data from 84 genotypes in the study of Koprivova et al. (2014) led to the discovery of a number of significantly associated markers, indicating a genetic component of leaf anion concentration traits. An updated, diversity population of 383 genotypes of *B. napus* has since been compiled and RNA-sequenced (Thomas et al., 2016; Havlickova et al., 2017). This forms an excellent foundation for Associative Transcriptomics (Harper et al., 2012), a technique that focuses on the analysis of transcribed sequences across diversity populations to identify high-resolution loci influencing complex traits. The use of RNA sequence data allows the development of markers based on both single-nucleotide polymorphisms (SNPs) and transcript abundance (gene-expression markers; GEMs). Gene expression levels may be particularly important in the control of traits in polyploid species such as *B. napus* in which gene duplication may have led to unequal expression (Adams et al., 2003). This technique has recently been used to dissect the genetic control of leaf Ca and Mg accumulation in *B. napus* (Alcock et al., 2017). Multiple genetic loci were highly associated with leaf Ca and Mg concentration, and numerous closely linked genes were identified as suitable candidates for their control. *Arabidopsis thaliana* plants mutated in orthologs of candidate genes were shown to have perturbed nutrient concentration traits, thus providing further evidence for their role in trait control and indicating the efficacy of this approach.

In this study, variation in leaf ${\text{NO}}_{3}^{-}$ concentration across a diversity population of 383 genotypes of *B. napus* was characterized by ion chromatography. This is likely to reflect most of the species-wide variation in this trait. Ion chromatography presented a high-throughput method suitable for rapid processing of the large number of samples associated with using a diversity population. As the major source of N to crops grown in aerobic soils, ${\text{NO}}_{3}^{-}$ is also likely to be a suitable measure of N status in *B. napus*. Leaf ${\text{NO}}_{3}^{-}$ data generated here, along with leaf P and K concentration data previously described in the same population (Thomas et al., 2016), was then utilized in Associative Transcriptomics analyses to identify single nucleotide polymorphisms (SNPs) and gene expression markers (GEMs) associated with these traits, as well as allelic variants which differ in ${\text{NO}}_{3}^{-}$, P and K accumulation, and genes in linkage disequilibrium (LD) likely to be responsible for their control.

## Materials and methods

### Growth of plant material

This study makes use of the Renewable Industrial Products from Rapeseed (RIPR) diversity population of inbred lines of *Brassica napus* genotypes (Thomas et al., 2016). A subset of 383 genotypes were selected, comprising 169 winter-, 123 spring-, and 11 semiwinter-oilseed rape (OSR), 27 swede, six fodder, three kale and 44 of unspecified growth types. All plant growth took place at the Sutton Bonington Campus of the University of Nottingham (52°49′58.9″N, 1°14′59.2″W) in compost in a designed experiment in a polytunnel as described previously (Thomas et al., 2016). Briefly, seeds were all sown into a fine-grade, medium nutrient compost (N144, P73, K239 ppm added; pH 5.3-6; Levington Seed & Modular + Sand -F2S; Everris Ltd., Ipswich, United Kingdom) in propagation trays and allowed to establish in a glasshouse with venting set to 15°C and supplementary lighting used to maintain day lengths of 12 h, for approximately 12 weeks. Plants were then transferred to individual 5 L pots containing a coarse, medium nutrient compost (N200, P150, K200 ppm added; pH 5.3-5.7; Levington C2; Scotts Professional, Ipswich, United Kingdom) and placed into single skinned polytunnels with no supplementary lighting or heating. Compost nutrient concentrations were deemed sufficient for plant growth prior to leaf sampling. Pots were arranged in a randomized block design of five replicate blocks, each split into 12 sub-blocks with 36 pots randomly allocated within. Sixteen reference genotypes were included within each replicate block to allow more accurate normalization. Pots were watered by automatic irrigation three times daily.

### Characterization of leaf nitrate, phosphorus and potassium concentrations

Leaves were sampled from all plants at the rosette stage (typically 6–8 true leaves showing) approximately 20 weeks after sowing as described previously (Thomas et al., 2016). This was after a period of vernalisation of all plants, but prior to stem extension in preparation for flowering, which enabled as close synchronization of growth stages as reasonably possible between genotypes of different growth habits (winter, spring OSR, etc.). Typically three leaves were taken from each plant and freeze dried for 48–60 h (CHRIST Alpha 2–4 LD freeze dryer; Martin Christ Gefriertrocknungsanlagen GmbH, Osterode, Germany). For each plant, all sampled leaves were pooled and homogenized in liquid N_2_ using a pestle and mortar and stored at −80°C prior to analyses. Since plants typically only had 6–8 true leaves at sampling stage, and sampling took place prior to stem extension in preparation for flowering, this is likely to reflect total shoot nutrient status. Leaf ${\text{NO}}_{3}^{-}$ concentration was measured using an ion chromatography (IC) system, following minor variations to the method described in Herschbach et al. (2000). For each dried, homogenized sample, 20 mg was weighed and transferred to a 1.5 mL Eppendorf tube (Eppendorf AG, Hamburg, Germany), to which 1.5 mL of Milli-Q water (18.2 MΩ cm; Fisher Scientific UK Ltd, Loughborough, United Kingdom) containing 20 mg of insoluble polyvinylpolypyrrolidone (PVPP) was added. This was incubated at 4°C for 60 min with frequent mixing, then at 90°C for 15 min in a water bath, prior to centrifugation at 5,000 RPM for 15 min at 4°C in an Eppendorf 5180 centrifuge (Eppendorf). The resulting clear supernatant was transferred into a 0.5 mL anion sample tube (PolyVials; Thermo Fisher Scientific). This was then analyzed by anion exchange chromatography using a Dionex™ ICS-1100 Ion Chromatography System, using IonPac AS14 analytical columns (Thermo Scientific, Waltham, MA, United States). Flow rate was 1.4 mL min^−1^ and temperature was set to 30°C. For each sample, injection volume was 50 μL. Eluents used were Na_2_CO_3_ (Fisher) and NaHCO_3_ (Fisher) at 2.7 mM and 1.8 mM, respectively, prepared in 2 L deionised water. Standards used were sodium fluoride, sodium chloride, potassium nitrate, potassium di-hydrogen orthophosphate, potassium sulfate, and di-sodium DL-malate prepared to 1,000 ppm then diluted to between 10 and 150 ppm across six standard samples. The instrument was primed for 20 min prior to analysis. Leaf P and K concentrations in all genotypes were previously characterized by inductively coupled plasma-mass spectrometry (ICP-MS; Thomas et al., 2016). Briefly, a further 200 mg of dry, homogenous sample was digested in 2 mL 70% v/v Trace Analysis Grade HNO_3_, 1 mL Milli-Q water, and 1 mL H_2_O_2_ in a microwave system (Multiwave 3,000; Anton Paar GmbH, Graz, Austria). Leaf digestates were diluted 1-in-5 using Milli-Q water, and then analyzed by ICP-MS with operational modes including (i) a helium collision-cell (He-cell) with kinetic energy discrimination to remove polyatomic interferences, (ii) standard mode (STD) in which the collision cell was evacuated, and (iii) a hydrogen collision-cell (H_2_-cell).

The relative contribution of genotypic and non-genotypic variance components underlying variation in leaf ${\text{NO}}_{3}^{-}$, P and K concentration was calculated using a REML procedure in GenStat (17th Edition, VSN International Ltd, Hemel Hempstead, United Kingdom). For leaf ${\text{NO}}_{3}^{-}$ concentration, genotype and experimental sources of variation were classed as random factors according to the model [IC_analysis_run_number + (genotype^*^replicate)]. For leaf P and K concentration traits, a further model was used [replicate + (replicate/sub-block) + genotype + (replicate/genotype)]. In the former model (for leaf ${\text{NO}}_{3}^{-}$ concentration), sub-block aliased with IC_analysis_run_number due to the order of sample analysis, and hence was largely accounted for without being specifically included in the model. Similarly, polytunnel was taken into account by replicate in each of the two models, since replicates were organized by distinct blocks within the two polytunnels used. For each analysis, genotype was subsequently added as a fixed factor to estimate normalized genotype-means. Standard deviations between genotypes within each of six defined crop habits (spring-, winter- and semiwinter-OSR, fodder, kale, and swede) as well as pairwise, Pearson correlation coefficients between leaf ${\text{NO}}_{3}^{-}$, P and K concentration data and two-sided tests of correlations against zero were calculated in GenStat (17th Edition).

Leaf nitrate, phosphorus and potassium normalized genotype-means utilized in this study are included in Supplementary Table 1 and at the Brassica Information Portal (BIP; https://bip.earlham.ac.uk; The Earlham Institute, Norwich, United Kingdom). Leaf nitrate concentration data as well as previously analyzed leaf mineral concentration data including phosphorus and potassium data used here are also available at DOIs 10.5281/zenodo.1321978 and 10.5281/zenodo.59937 respectively.

### Transcriptome sequencing, SNP identification and quantification of transcript abundance

Plant growth conditions, sampling techniques, RNA extraction, quality checking and Illumina transcriptome sequencing were described previously (He et al., 2017). For each genotype, RNA-sequence data was mapped onto ordered *Brassica* A and C genome-based pan-transcriptomes developed by He et al. (2015) using methods described by Bancroft et al. (2011) and Higgins et al. (2012). SNP positions with a read depth below 10, base call quality below Q20, or missing data above 25% among all genotypes, were excluded from alignment, as were SNP positions for which >3 alleles were called. Across the 383 genotype panel, 46,307 single SNPs and 309,229 hemi-SNPs were detected and scored. Transcript abundance was quantified and normalized as reads per kb per million aligned reads (RPKM) for each genotype. Of the 116,098 coding DNA sequence (CDS) models in the pan-transcriptome, significant expression (mean >0.4 RPKM) was detected for 53,889. Transcriptome sequences are deposited within the Sequence Read Archive (Leinonen et al., 2011) under accession number PRJNA309367. Clustering of genotypes based on SNP data was previously carried out, and a dendogram visualization can be found in Havlickova et al. (2017).

### Associative transcriptomics

Association analyses using SNPs and GEMs were carried out in R 3.2.0 (R Core Team, 2015) as previously described by Harper et al. (2012) with the modifications recently described by Havlickova et al. (2017). Inference of population structure by Q-matrix was obtained by Population Structure Inference using Kernel-PCA and Optimization (PSIKO; Popescu et al., 2014; highest likelihood subpopulation *k* = *2*). SNP-based analyses were performed using a compressed mixed linear model approach (Zhang et al., 2010) implemented in the GAPIT R package (Lipka et al., 2012). GEM-based analyses were performed using fixed-effect linear modeling in R with RPKM values and PSIKO-inferred Q-matrix data as the explanatory variables and trait score the response variable. Coefficients of determination (R^2^), constants and significance values were output for each regression. Genomic control (Devlin and Roeder, 1999) was applied to the GEM analysis when the genomic inflation factor was observed to be >1 to correct for spurious associations. Manhattan plots reporting –log_10_*p*-values for each marker for each trait were generated using graph functions in R. SNPs with low second allele frequency (< 0.01) were filtered from the dataset prior to plot generation. In total 256,397 SNPs and 53,889 GEMs were plotted. SNP markers for which genetic mapping to the appropriate genome could not be confirmed due to sequence similarity are plotted in gray, and appear in both *Brassica* A and C sub-genomes. False Discovery Rate (FDR; Benjamini and Hochberg, 1995) and Bonferroni (Dunn, 1961) corrections were used to set significance thresholds at *p* < 0.05. QQ plots for all association analyses are included as Supplementary Figures 2–7.

### Candidate gene selection

Genome browsers comprising sequences of *B. rapa* (A genome, Chiifu-401-41; Wang et al., 2011) and *B. oleracea* (C genome, TO1000DH3; Parkin et al., 2014) at Ensembl Plants (Kersey et al., 2016) as well as ordered *Brassica* pan-transcriptome data (He et al., 2015) were used to determine genes that fell within visually-determined association peaks and were within estimated LD decay (~1–2 cM on average; Ecke et al., 2010) of SNPs associated with measured traits. All genes that fulfilled these requirements were considered in candidate gene selection. Predicted gene function, inferred using ortholog annotations from closely related *Arabidopsis thaliana* held by The Arabidopsis Information Resource (TAIR; Huala et al., 2001), gene expression data at The Bio-Analytic Resource for Plant Biology (Waese and Provart, 2017) and ionomic data at the Purdue Ionomics Information Management System (PIIMS; Baxter et al., 2007) was used to select for genes most likely contributing to the control of leaf ${\text{NO}}_{3}^{-}$, P and/or K concentration. For candidate genes derived from GEM analyses, only genes corresponding to GEMs directly associated with the measured traits were considered.

### Lead-marker allelic effects on leaf nitrate, phosphorus and potassium concentration

For SNP-based association analyses, the allelic effects of lead-markers on leaf ${\text{NO}}_{3}^{-}$ and P concentration traits were determined. Lead-markers were defined as the most highly associated SNP markers within visually-determined association peaks for a given trait that had a second allele frequency above 0.01 and had been correctly mapped to the appropriate *B. napus* sub-genome. For each lead-marker, leaf ${\text{NO}}_{3}^{-}$ or P concentration data were plotted separately for each allele at the SNP locus. Box plots representing allelic effects were created in SigmaPlot (13th Edition, Systat Software Inc., San Jose, CA, United States). For leaf K concentration, scatter plots representing marker expression effects on the trait were plotted for all markers with –log_10_*p*-values greater than the FDR corrected significance threshold (*p* = 0.05) as well as markers corresponding to described candidate genes (Table 1). Scatter plots and coinciding linear regression lines were generated in SigmaPlot. *r*^2^-values and respective significance values were calculated in GenStat.

**Table 1:** Candidate genes within estimated linkage disequilibrium decay of lead-markers associated with leaf nitrate, phosphorus, and/or potassium concentration.

**Trait**	**Candidate gene**	***Arabidopsis ortholog***	***E*-value**	**Putative function**	**Linkage group**	**Lead-marker ID**
Leaf ${\text{NO}}_{3}^{-}$ concentration	Cab024292.1	AT4G24020	0.0	NIN-LIKE PROTEIN 7; involved in regulation of nitrate assimilation	A1	Cab024343.4:2028:T
Leaf ${\text{NO}}_{3}^{-}$ concentration	Cab002472.4	AT5G10140	5 × 10 ^−33^	FLOWERING LOCUS C; MADS-box transcription factor family protein	A3	Cab003105.1:423:C
Leaf ${\text{NO}}_{3}^{-}$ concentration	Cab035003.1	AT2G37360	7 × 10^−312^	ATP-BINDING CASSETTE G2; required for root suberin synthesis	A4	Cab034977.1:588:G
Leaf ${\text{NO}}_{3}^{-}$ concentration	Cab034940.1	AT2G38290	2 × 10^−186^	AMMONIUM TRANSPORTER 2	A4	Cab034977.1:588:G
Leaf ${\text{NO}}_{3}^{-}$ concentration	Cab000535.1	AT3G53510	5 × 10^−167^	ATP-BINDING CASSETTE G20; required for root suberin synthesis	A9.1	Cab000410.2:940:G
Leaf ${\text{NO}}_{3}^{-}$ concentration	Cab014282.1	AT1G08090	5 × 10^−252^	NITRATE TRANSPORTER 2.1	A9.2	Cab014338.1:34:T
Leaf ${\text{NO}}_{3}^{-}$ concentration	Cab014283.1	AT1G08090	3 × 10^−291^	NITRATE TRANSPORTER 2.1	A9.2	Cab014338.1:34:T
Leaf ${\text{NO}}_{3}^{-}$ concentration	Cab017152.1	AT5G60780	3 × 10^−270^	NITRATE TRANSPORTER 2.3	A10	BnaA10g13370D:657:T
Leaf ${\text{NO}}_{3}^{-}$ concentration	Cab017154.1	AT5G60770	3 × 10^−286^	NITRATE TRANSPORTER 2.4	A10	BnaA10g13370D:657:T
Leaf ${\text{NO}}_{3}^{-}$ concentration	Bo1g037430.1	AT4G24020	0.0	NIN-LIKE PROTEIN 7; involved in regulation of nitrate assimilation	C1	Bo1g036990.1:1395:C
Leaf ${\text{NO}}_{3}^{-}$ concentration	Bo4g145580.1	AT5G40890	0.0	CHLORIDE CHANNEL A; involved in nitrate accumulation in vacuoles	C4	Bo4g143900.1
Leaf ${\text{NO}}_{3}^{-}$ concentration	Bo8g082890.1	AT3G53510	0.0	ATP-BINDING CASSETTE G20; required for root suberin synthesis	C8	Bo8g082080.1:192:A
Leaf ${\text{NO}}_{3}^{-}$ concentration	Bo9g147020.1	AT5G60780	2 × 10^−173^	NITRATE TRANSPORTER 2.3	C9	Bo9g143680.1:1029:T
Leaf ${\text{NO}}_{3}^{-}$ concentration	Bo9g146990.1	AT5G60770	8 × 10^−164^	NITRATE TRANSPORTER 2.4	C9	Bo9g143680.1:1029:T
Leaf ${\text{NO}}_{3}^{-}$ concentration	Bo9g147000.1	AT5G60770	0.0	NITRATE TRANSPORTER 2.4	C9	Bo9g143680.1:1029:T
Leaf P concentration	Cab013031.3	AT4G16480	2 × 10^−297^	INOSITOL TRANSPORTER 4	A1	Cab013058.2:573:G
Leaf P concentration	Cab012953.1	AT4G17230	7 × 10^−252^	SCARECROW-LIKE 13; required for the regulation of hypocotyl elongation	A1	Cab013058.2:573:G
Leaf P concentration	Cab023424.2	AT3G08500	1 × 10^−137^	MYB83; transcription factor required for activation of lignin synthesis	A5	Cab023469.1:384:C
Leaf P concentration	Cab022857.1	AT3G54700	8 × 10^−262^	PHOSPHATE TRANSPORTER 1.7	A6	Cab022858.1:2706:G
Leaf P concentration	Cab022780.1	AT1G50420	1 × 10^−131^	SCARECROW-LIKE 3; involved in control of gibberellin-mediated plant growth	A6	Cab022858.1:2706:G
Leaf P concentration	Cab022771.1	AT1G50600	1 × 10^−265^	SCARECROW-LIKE 5; transcription factor likely to be involved in plant growth	A6	Cab022858.1:2706:G
Leaf P concentration	Bo2g020730.1	AT5G20240	6 × 10^−116^	PISTILLATA; MADS-box transcription factor involved in floral development	C2	Bo2g017990.1:1221:G
Leaf P concentration	Bo2g018220.1	AT5G19600	0.0	SULFATE TRANSPORTER 3.5	C2	Bo2g017990.1:1221:G
Leaf P concentration	Bo4g182590.1	AT2G34440	1 × 10^−64^	AGAMOUS-LIKE 29; transcription factor likely involved in floral development	C4	Bo4g182560.1:642:G
Leaf P concentration	Bo4g182170.1	AT2G33770	2 × 10^−125^	PHOSPHATE 2 / UBC24; involved in phosphate starvation response	C4	Bo4g182560.1:642:G
Leaf P concentration	Bo4g185840.1	AT2G38940	4 × 10^−116^	PHOSPHATE TRANSPORTER 1.4	C4	Bo4g182560.1:642:G
Leaf P concentration	Bo4g184590.1	AT2G35740	6 × 10^−137^	INOSITOL TRANSPORTER 3	C4	Bo4g182560.1:642:G
Leaf P concentration	Bo8g108330.1	AT1G12240	0.0	BFRUCT4; plays a role in mobilizing sucrose to sink organs and in root elongation	C8	Bo8g108620.1:687:A
Leaf P concentration	Bo8g108240.1	AT1G12360	2 × 10^−54^	KEULE; regulates cytokinesis related vesicle trafficking and root hair formation	C8	Bo8g108620.1:687:A
Leaf P concentration	Bo8g108070.1	AT1G12560	1 × 10^−111^	EXPANSIN A7; ex pressed in root hair cells and involved in elongation	C8	Bo8g108620.1:687:A
Leaf P concentration	Bo8g107860.1	AT1G12950	2 × 10^−96^	ROOT HAIR SPECIFIC 2; positively mediates root hair elongation	C8	Bo8g108620.1:687:A
Leaf P concentration	Bo9g166650.1	AT5G13580	0.0	ATP-BINDING CASSETTE G6; required for root suberin synthesis	C9.1	Bo9g166760.1:437:A
Leaf P concentration	Bo9g166210.1	AT5G14040	4 × 10^−143^	PHOSPHATE TRANSPORTER 3.1	C9.1	Bo9g166760.1:437:A
Leaf P concentration	Bo9g173370.1	AT5G10140	9 × 10^−73^	FLOWERING LOCUS C; MADS-box transcription factor family protein	C9.2	Bo9g173750.1:963:A
Leaf K concentration	Cab024257.1	AT4G23640	0.0	POTASSIUM TRANSPORTER 3; also required for tip growth of root hairs	A1	GEM_Cab024257.1
Leaf K concentration	Cab001225.1	AT2G04850	2 × 10^−200^	Au × in-responsive family protein	A3	GEM_Cab001225.1
Leaf K concentration	Cab002494.2	AT2G30320	3 × 10^−207^	Pseudouridine synthase family protein	A3	GEM_Cab002494.2
Leaf K concentration	Cab037725.3	AT1G05940	9 × 10^−237^	CATIONIC AMINO ACID TRANSPORTER 9	A9	GEM_Cab037664.1
Leaf K concentration	Cab007712.1	AT5G10740	2 × 10^−151^	Protein phosphatase 2C family protein	A10	GEM_Cab007712.1
Leaf K concentration	Bo9g003730.1	AT4G01080	5 × 10^−50^	TRICHOME BIREFRINGENCE-LIKE 26	C9	GEM_Bo9g003730.1
Leaf K concentration	Bo9g002280.1	AT4G00360	0.0	CYTOCHROME P450, FAMILY 86, SUBFAMILY A, POLYPEPTIDE 2	C9	GEM_Bo9g002280.1
Leaf K concentration	Bo9g001030.1	AT1G05940	6 × 10^−143^	CATIONIC AMINO ACID TRANSPORTER 9	C9	GEM_Bo9g002280.1
Leaf K concentration	Bo9g171810.1	AT5G10740	3 × 10^−47^	Protein phosphatase 2C family protein	C9	GEM_Bo9g171810.1

*Putative functions obtained from annotation data at The Arabidopsis Information Resource (Huala et al., 2001). Sequences of both Brassica and A. thaliana genes available at Ensembl Plants (Kersey et al., 2016). Expect (E) value between coding DNA sequences of Brassica candidate genes and A. thaliana orthologs generated using BLAST functions at Ensembl Plants*.

## Results

### Oilseed rape varieties have the highest mean leaf concentrations of nitrate, phosphorus and potassium

**Figure 1:**
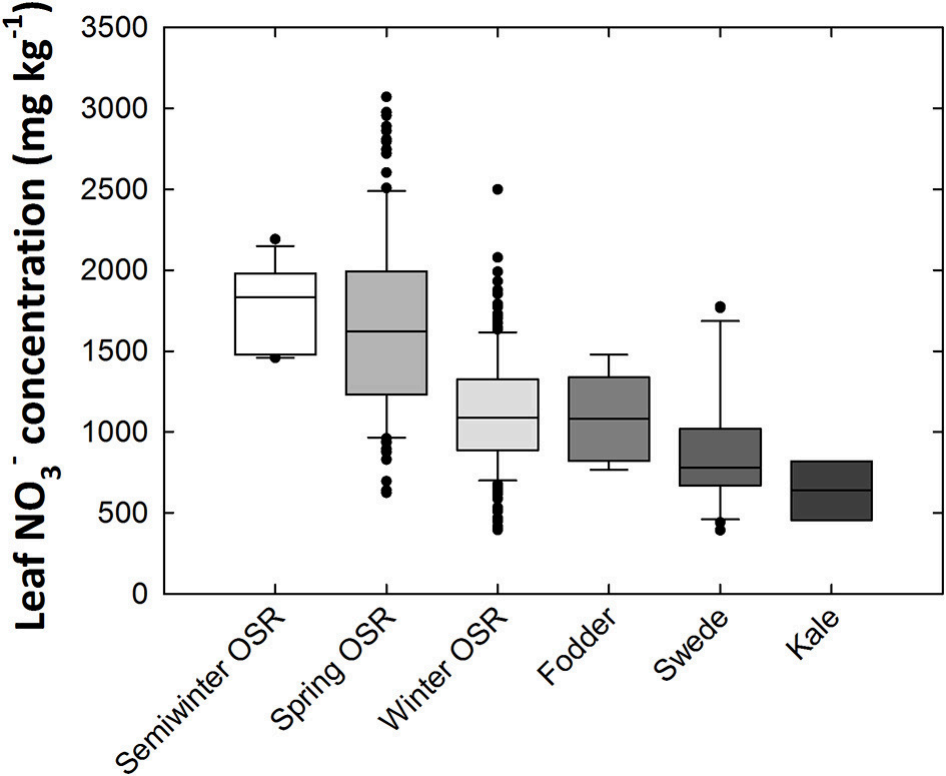
Leaf nitrate concentrations of *Brassica napus* plants grown in compost. Data are means of 339 genotypes for which crop growth type had been assigned including 169 winter-, 11 semiwinter-, and 123 spring-oilseed rape (OSR), 27 swede, six fodder and three kale types. Boxes represent the mid two quartiles with the median drawn; whiskers above and below the boxes indicate the 90 and 10th percentiles with any outliers shown. Boxes organized by mean leaf nitrate concentration between crop growth types from highest (left) to lowest (right).

Mean leaf ${\text{NO}}_{3}^{-}$ concentration varied 8.34-fold between genotypes, ranging from 368 to 3070 mg kg^−1^ dry weight (DW; Figure 1). When compared between crop types, the highest average leaf ${\text{NO}}_{3}^{-}$ concentrations were observed in oilseed rape (OSR) varieties, particularly semiwinter and spring OSR types. Ranked highest to lowest in terms of leaf ${\text{NO}}_{3}^{-}$ concentration, the crop-types fall in the order of semiwinter OSR, spring OSR, winter OSR, fodder, swede, and kale, with average leaf ${\text{NO}}_{3}^{-}$ concentrations of 1,775, 1654, 1,129, 1,090, 890, and 637 mg kg^−1^ DW, respectively. Standard deviations within these groups were 255, 556, 363, 282, 377, and 182, respectively. Large amounts of variation were observed within both winter and spring OSR types; 6.34- and 4.92-fold variation, respectively (Figure 1). Leaf P and K concentration data were reported previously (Thomas et al., 2016). Briefly, leaf P and K concentrations varied 1.98- and 2.07-fold from 3,290 to 6,526 mg kg^−1^ DW and 26,228 to 54,329 mg kg^−1^ DW, respectively, between all 383 genotypes. Mean leaf P concentration was highest in spring OSR types, followed by fodder, semiwinter OSR, winter OSR, kale, and finally swede types. Mean leaf K concentration was highest in semiwinter OSR, followed by spring OSR, winter OSR, swede, fodder, and finally kale types. Pearson correlation coefficient between leaf ${\text{NO}}_{3}^{-}$ and K was 0.45; between leaf ${\text{NO}}_{3}^{-}$ and P was 0.32; and between leaf P and leaf K was 0.25 (all *p* < 0.001). The range of concentrations of each nutrient followed approximately a normal distribution across the diversity population (Supplementary Figure 1).

### Genes controlling root suberin synthesis and nitrate and ammonium transport are within LD decay of markers associated with leaf nitrate concentration

**Figure 2:**
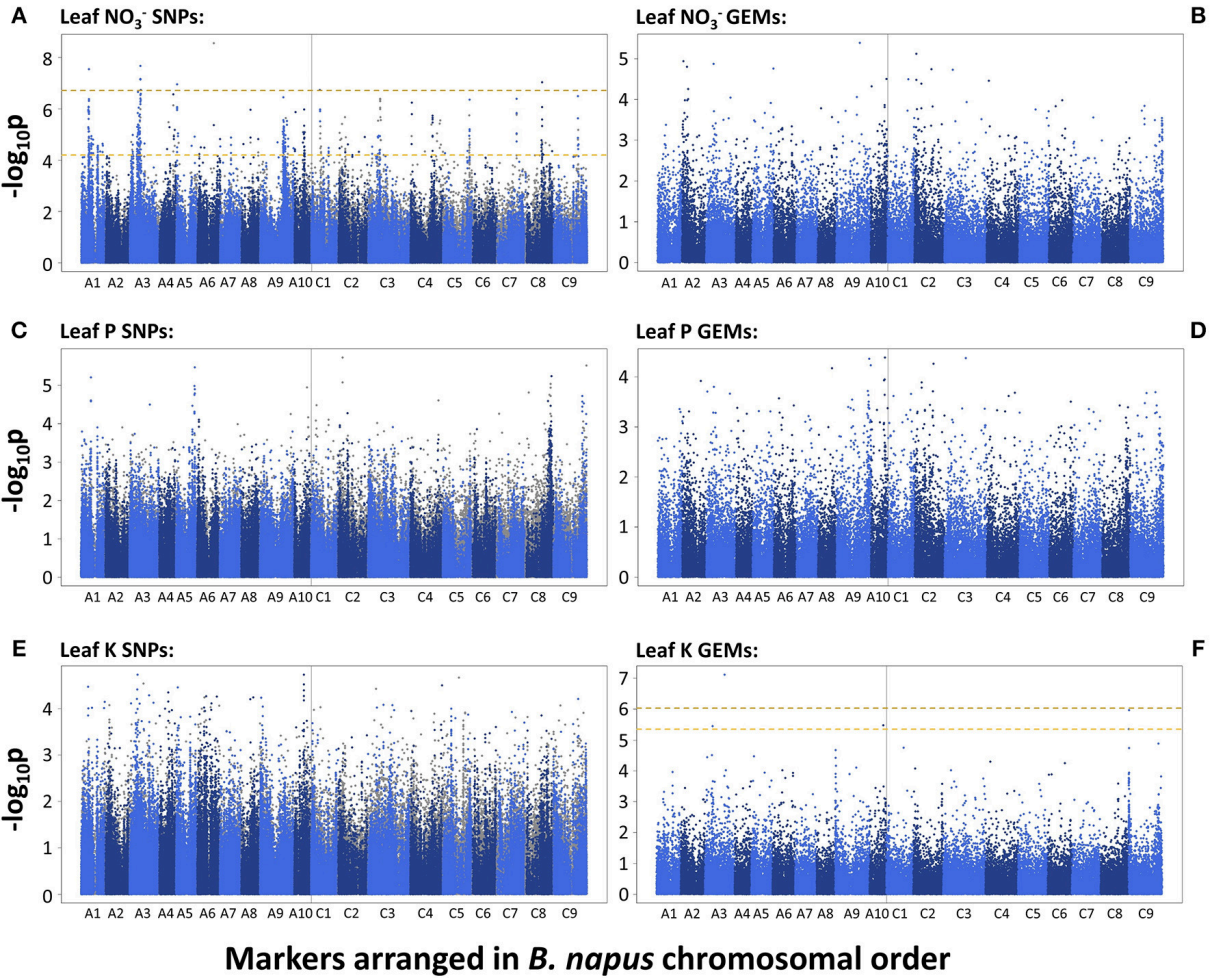
–log_10_*p*-values of SNPs and GEMs associated with leaf nitrate concentration (**A**,**B**, respectively), leaf phosphorus concentration (**C**,**D**, respectively) and leaf potassium concentration (**E**,**F**, respectively) in order of markers within the *Brassica napus* pan-transcriptome. Upper, gold, dashed line represents Bonferroni corrected significance threshold; lower, yellow, dashed line represents FDR corrected significance threshold (*p* = 0.05).

A total of 455 and 10 SNP markers were significantly associated with leaf ${\text{NO}}_{3}^{-}$ concentration after FDR and Bonferroni correction respectively (*p* = 0.05). This was reduced to 415 and 9 SNP markers, respectively, after filtering out markers with low second allele frequency (SAF; < 0.01). The majority of associated markers fell within visually-determined association peaks. The two most defined association peaks are located on chromosomes A1 and A3 and co-localize with one and four of the 10 SNPs above the Bonferroni corrected significance threshold, respectively (*p* = 0.05). Further, notable association peaks are located on chromosomes A4, A9, A10, C1, C3 C4, C5, C8, and C9 (Figure 2A). There were no GEMs that fell above FDR or Bonferroni corrected significance thresholds. However, less distinct association peaks were visually-determined on chromosomes A2, C2, and C9 (Figure 2B). By searching genetic loci within linkage disequilibrium (LD) decay (~1–2 cM on average; Ecke et al., 2010) of SNP-based association peaks associated with leaf ${\text{NO}}_{3}^{-}$ concentration, 15 candidate genes were identified (Table 1). These were orthologous to nine unique *Arabidopsis thaliana* genes, with multiple, paralogous copies of some candidate genes within LD decay of associated markers. Within LD of the association peak on chromosome A1 lies Cab024292.1, an ortholog of *A. thaliana* AT4G24020.1. This gene encodes NIN (nodule inception)-LIKE PROTEIN 7 (NLP7), a putative transcription factor thought to be responsible for sensing and responding to exogenous nitrate concentrations (Castaings et al., 2009). A further ortholog of *NLP7* was found within the syntenous region of chromosome C1 (Bo1g037430.1). Within the association peak on chromosome A3 lies Cab002472.4, an ortholog of *A. thaliana* AT5G10140.4. This encodes FLOWERING LOCUS C (*FLC*), a transcription factor responsible for repressing flowering prior to sufficient cold treatment (Sheldon et al., 2000). The most highly associated GEM on chromosome A3 also corresponds to Cab002472.4. Further candidate genes include seven members of the NITRATE TRANSPORTER 2 (*NRT2*) gene family (paralogous copies of *NRT2.1*, Cerezo et al., 2001; *NRT2.3*, Orsel et al., 2002; *NRT2.4*, Kiba et al., 2012), three genes encoding ABC transporters thought to be required for suberin synthesis in roots (*ABCG2*; and paralogous copies of *ABCG20*; Yadav et al., 2014), a gene encoding a high-affinity ammonium transporter (*AMT2*; Sohlenkamp et al., 2002), and a chloride channel (*CLCa*) which is thought to be responsible for ${\text{NO}}_{3}^{-}$ accumulation in vacuoles (De Angeli et al., 2006; Table 1).

**Figure 3:**
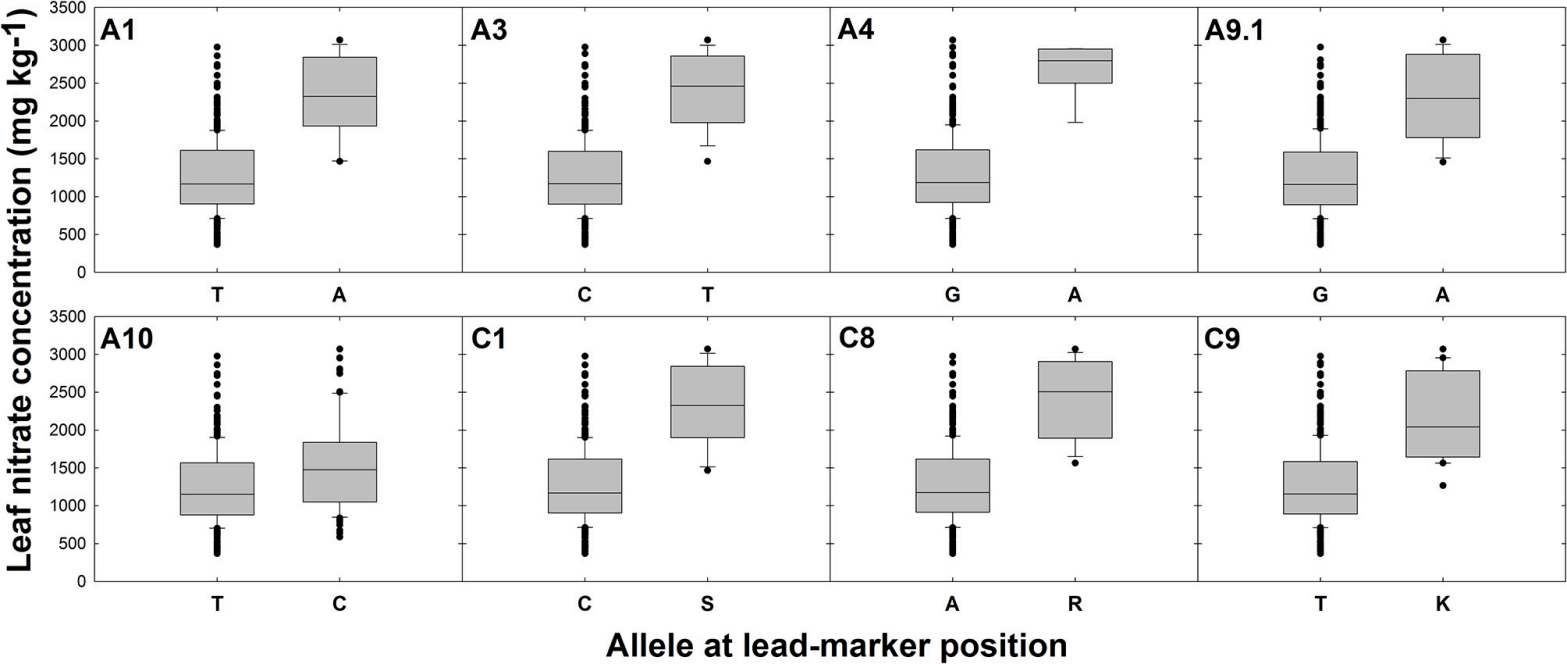
Effects of reference allele and most frequent allelic variant at lead-marker loci on leaf nitrate concentration. Each panel refers to a specific association peak; lead-markers at each peak are listed in table 1. Boxes represent the mid two quartiles with the median drawn; whiskers above and below the boxes indicate the 90 and 10th percentiles with any outliers shown. A, T, G, and C alleles represent adenine, thymine, guanine and cytosine nucleotide calls, respectively. S, R, and K alleles represent Strong (C or G), purine (A or G) or Keto (C or T) nucleotide calls, respectively, thus called due to unresolvable variation in closely related genes. Differences in leaf nitrate concentration between alleles at each loci significant at *p* < 0.001.

Differences in gene expression between oilseed crop-type groups were observed for five candidate genes. Specifically, elevated transcript abundance of Cab034940.1 (*AMT2*) and Bo1g037430.1 (*NLP7*) was observed in semiwinter compared to spring and winter OSR genotypes (*p* < 0.01) and lower transcript abundance of Bo4g145580.1 (*CLCa*) and both Cab017152.1 and Bo9g147020.1 (both homeologous copies of *NTR2.3*) was observed in winter compared to spring OSR genotypes (*p* < 0.05, 0.001, and 0.001, respectively). For lead-markers within selected association peaks, genotype-specific allelic variants in the RIPR diversity population were identified and leaf nitrate concentration between variant and reference alleles were compared. The allelic variant was defined as the second most frequent allele in the population after the reference base call. Lead-markers for each association peak investigated are summarized in Table 1. For lead-markers within association peaks on chromosomes A1, A3, A4, A9 (labeled A9.1 as two association peaks referred to in this chromosome in Table 1), A10, C1, C8, and C9, the allelic variant was associated with a 1.86-, 1.93-, 2.06-, 1.84-, 1.24-, 1.85-, 1.92-, and 1.73-fold increase in leaf ${\text{NO}}_{3}^{-}$ concentration respectively (Figure 3; all *p* values < 0.001).

### Genes controlling root-hair development among candidates for leaf phosphorus concentration

**Figure 4:**
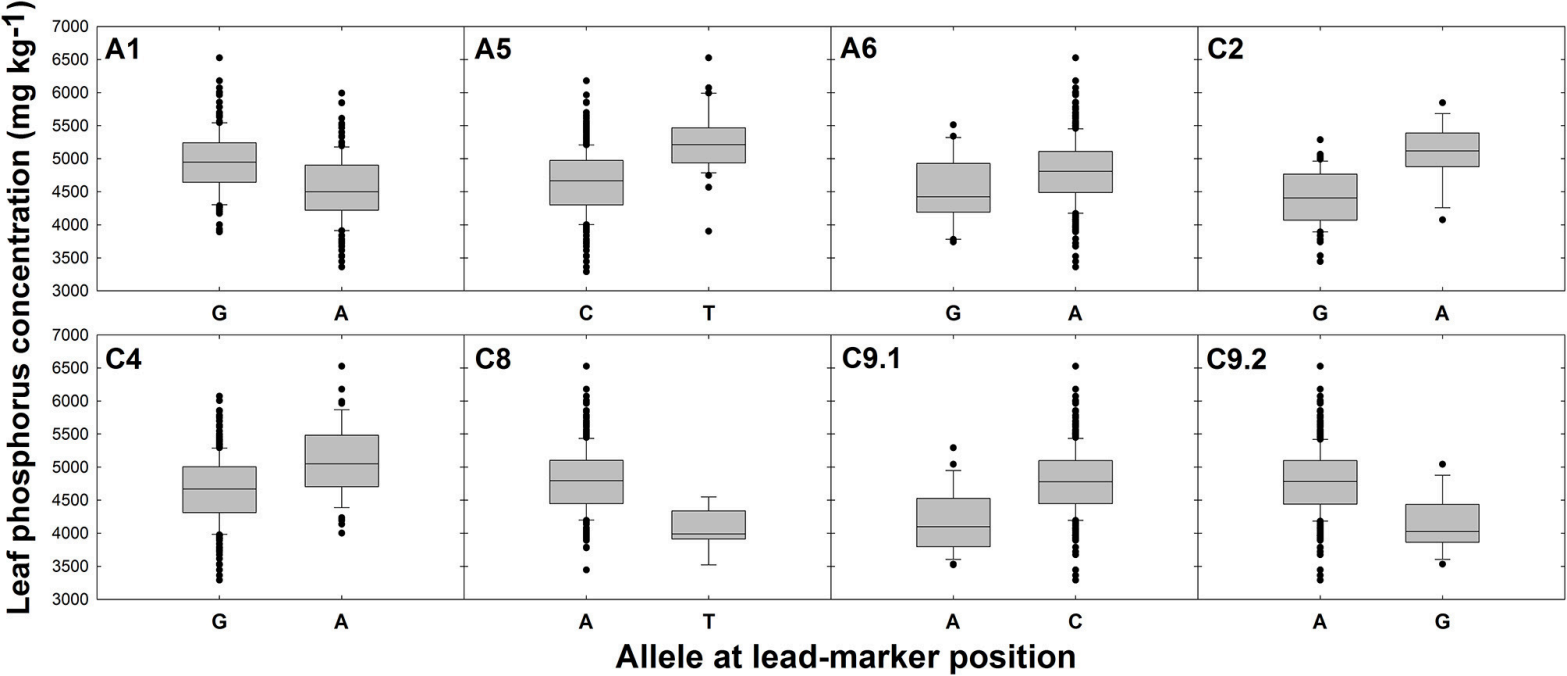
Effects of reference allele and most frequent allelic variant at lead-marker loci on leaf phosphorus concentration. Each panel refers to a specific association peak; lead-markers at each peak are listed in table 1. Boxes represent the mid two quartiles with the median drawn; whiskers above and below the boxes indicate the 90 and 10th percentiles with any outliers shown. A, T, G, and C alleles represent adenine, thymine, guanine and cytosine nucleotide calls, respectively. Differences in leaf phosphorus concentration between alleles at each loci significant at *p* < 0.001 except locus on chromosome A6; *p* = 0.010.

There were no SNPs or GEMs that associated with leaf P concentration above the level of Bonferroni or FDR corrected significance thresholds. However, a number of SNP markers had notably higher –log_10_*p* values than the majority of markers, and many of these formed visually-determinable association peaks (Figure 2C). The most distinct of these are present on chromosomes A1, A5, C8, and C9. Further, less prominent association peaks were detected on chromosomes A6, C2, and C4. A total of 19 candidate genes were identified within LD decay of associated markers at these loci (Table 1). Most notable candidate genes are four genes involved in root or root hair development on chromosome C8. These are orthologous to *A. thaliana* AT1G12240.1 (*BFRUCT4*), AT1G12360.1 (*KEULE*), AT1G12560.1 (*EXPANSIN A7*), and AT1G12950.1 (*ROOT HAIR SPECIFIC 2*). An additional outstanding candidate is a Bo9g166650, an ortholog of *A. thaliana* AT5G13580.1. This encodes an ABC transporter of the same family as those within LD of markers associated with leaf ${\text{NO}}_{3}^{-}$ concentration and this member is also thought to be required for root suberin synthesis. Further candidates include three genes encoding SCARECROW-LIKE proteins (3, 5, and 13) which are transcription factors involved in the control of developmental processes during the plant life cycle, as well as three genes encoding flowering time regulators, six transporter-protein encoding genes, a gene involved in response to low phosphate, and a transcription factor which may have a role in lignin synthesis (*MYB83*; Zhong and Ye, 2012; Table 1). It is also worthy of note that two of the GEMs most highly associated with leaf P concentration (chromosomes A10 and A3, ranked 1 and 11th, respectively; Figure 2D) correspond to distinct, orthologous copies of *A. thaliana* AT5G10140.4 (*FLC*). The latter of these is identical to the gene within LD of the association peak identified for leaf ${\text{NO}}_{3}^{-}$ concentration described above. For all eight loci within which candidate genes were identified, box plots were generated to visualize the effect of allelic variant at lead-marker position on leaf P concentration (Figure 4). For lead-markers within association peaks on chromosomes A5, A6, C2, C4, and the former peak on C9 (C9.1), the allelic variant was associated with a 1.13-, 1.06-, 1.15-, 1.09-, and 1.14-fold increase in leaf P concentration compared to the reference allele, respectively (*p* < 0.001, *p* = 0.010, *p* < 0.001, *p* < 0.001, *p* < 0.001, respectively). For lead-markers within association peaks on chromosomes A1, C8, and the latter peak on chromosome C9 (C9.2), the allelic variant was associated with a 1.09-, 1.18-, and 1.15-fold decrease in leaf P concentration compared to the reference allele (all *p* < 0.001).

### The most highly associated GEM for leaf potassium concentration corresponds to an auxin responsive family protein

**Figure 5:**
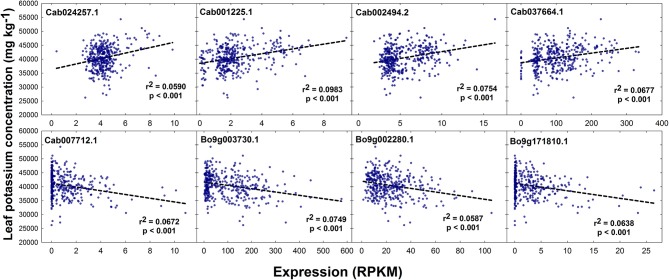
Effect of leaf transcript abundance of genes underlying candidate GEMs on leaf potassium concentration. Transcript abundance was quantified and normalized as reads per kb per million aligned reads (RPKM) for each genotype. *r*^2^ values and respective significance values calculated in GenStat are shown.

No SNPs, but a total of 1 and 5 GEMs were associated with leaf K concentration above the level of Bonferroni and FDR corrected significance thresholds, respectively, after genomic control (Figures 2E,F). The GEM above both the significance thresholds was located on chromosome A3 and corresponds to Cab001225.1, an ortholog of *A. thaliana* AT2G04850.1 which encodes an auxin-responsive family protein. Of the remaining four GEMs over the FDR corrected significance threshold, a further marker falls within chromosome A3, one within chromosome A10, and two within chromosome C9 (Figure 2F). Unlike results from SNP-based analyses, for which any gene within LD decay of associated markers could be the cause of the variation observed in the trait, genes underlying GEMs are more likely to be directly involved in trait control. For this reason, genes under all five of the aforementioned GEMs were considered as likely candidate genes (Table 1). However, two visually-determined association peaks were located toward the beginning of chromosomes A9 and C9, and hence genes within LD of these association peaks were also considered. Within LD decay of the more highly associated markers within these loci are the genes Cab037725.3 and Bo9g001030.1, both orthologous to *A. thaliana* AT1G05940.1, a gene that encodes a cationic amino acid transporter. Two further candidate genes with relatively high –log_10_*p*-values in the corresponding GEM were considered; Cab024257.1 and Bo9g171810.1. The former of these is orthologous to *A. thaliana* AT4G23640.1 which encodes a potassium transporter family protein. The latter is paralogous to the gene underlying the GEM over the FDR corrected significance threshold on chromosome A10. The effects of expression levels of the GEMs underlying each of these candidate genes is modeled in Figure 5. For GEMs underlying genes Cab024257.1, Cab001225.1, Cab002494.2 and Cab037664.1, an increase in expression was associated with an overall increase in genotype-specific mean leaf K concentration (*r*^2^ = 0.059, 0.098, 0.075, and 0.068 respectively, all *p* < 0.001). For GEMs underlying genes Cab007712.1, Bo9g003730.1, Bo9g002280.1 and Bo9g171810.1, an increase in expression was associated with an overall decrease in genotype-specific mean leaf K concentration (*r*^2^ = 0.067, 0.075, 0.059 and 0.064 respectively, all *p* < 0.001; Figure 5).

## Discussion

### Species-wide variation in leaf nitrate concentration indicates scope for breeding for increased N uptake and use efficiency via traditional means

Leaf ${\text{NO}}_{3}^{-}$ concentration varied over 8-fold among the 383 genotypes in the RIPR diversity population, indicating large amounts of species-wide variation. The variation observed here is lower than the 83-fold variation shown previously in a population of 84 similar genotypes of *B. napus* grown under field conditions (Koprivova et al., 2014). After converting FW measurements to DW for comparison, assuming a 90% leaf water content, genotypes in the study of Koprivova et al. (2014) had estimated minimum and maximum leaf ${\text{NO}}_{3}^{-}$ concentrations of 192 and 15,935 mg kg^−1^ DW, respectively. Hence, values from the present study occupy similar orders of magnitude to those measured previously. The lower variance between genotypes observed here is likely linked to the age of the plants, which were sampled after approximately 4 months of growth. In the earlier study of Koprivova et al. (2014), they were sampled after only 8 weeks. It could also be linked the growth conditions, which in the present study comprised compost-filled pots under controlled irrigation placed in polytunnels, unlike the field conditions used in the earlier study. Despite these differences, it is clear that large amounts of species-wide variation in shoot ${\text{NO}}_{3}^{-}$ concentration exist in *B. napus*, irrespective of growth condition or plant age. This knowledge could be applied in traditional breeding strategies for increasing N uptake and use efficiency, perhaps under low exogenous ${\text{NO}}_{3}^{-}$ conditions. It is worth bearing in mind that a low leaf ${\text{NO}}_{3}^{-}$ concentration may be indicative of a high N use efficiency. There are multiple definitions of N use efficiency including yield per unit of N input, amount of N in plant per unit of N input, and shoot weight per unit of shoot N (Good et al., 2004). If a crop has relatively low shoot concentrations of ${\text{NO}}_{3}^{-}$ but still yields highly, it may possess traits that allow it to prosper under lower exogenous ${\text{NO}}_{3}^{-}$ supply. Hence it is essential that genotype-specific yield be considered as well as leaf ${\text{NO}}_{3}^{-}$ concentration in selecting suitable varieties for breeding. It is also important to consider the limitations of sampling plants grown under only a single exogenous N supply at a single developmental time point, as performed in this study, in the assessment of variation in N use between genotypes. It is possible that further variation could be uncovered if plants were grown and sampled under N-limiting conditions. This could lead to the identification of genotypes suitable for breeding novel crop cultivars that are more vigorous in low N conditions than current, widely cultivated germplasm. It may also lead to the identification of genetic loci controlling leaf ${\text{NO}}_{3}^{-}$ accumulation under low exogenous supply, which may be particularly useful in more targeted breeding strategies.

### Identification of genes encoding nitrate and ammonium transporters within LD of leaf nitrate association peaks likely linked to altered expression between genotypes in the diversity population

A total of eight genes encoding putative ${\text{NO}}_{3}^{-}$ transporters and one gene encoding a putative ammonium (${\text{NH}}_{4}^{+}$) transporter were identified within LD decay of markers highly associated with leaf ${\text{NO}}_{3}^{-}$ concentration. Seven of the eight genes encoding ${\text{NO}}_{3}^{-}$ transporters are members of the NITRATE TRANSPORTER 2 (*NRT2*) family and fall within three distinct loci on chromosomes A9 (two orthologs of *Arabidopsis thaliana NRT2.1*), A10 (orthologs of *A. thaliana NRT2.3* and *NRT2.4*) and C9 (further orthologs of *A. thaliana NRT2.3* and *NRT2.4*; Table 1). The latter two loci are in regions of known shared sequence homology (Chalhoub et al., 2014), indicating that copies of these genes on both *B. napus* sub-genomes are likely to be important for leaf ${\text{NO}}_{3}^{-}$ accumulation. Each of these genes encode high-affinity ${\text{NO}}_{3}^{-}$ transporters, transcript abundances of which are high under low exogenous ${\text{NO}}_{3}^{-}$ concentrations (Orsel et al., 2002). *NRT2*.3 forms a highly interesting candidate, since transcript abundance of both paralogous copies discussed above is lower in winter compared to spring OSR genotypes. This may contribute to the typically lower leaf ${\text{NO}}_{3}^{-}$ concentrations observed in winter compared to spring OSR genotypes. Tissue specific gene expression analysis of *A. thaliana NRT2.3* revealed expression in both root and shoot tissue throughout developmental stages (Orsel et al., 2002). Intriguingly, whilst *A. thaliana NRT2.3* shows a nitrate inducible expression pattern in shoots, root gene expression appears to be constitutive (Okamoto et al., 2003). However, to our knowledge, specific, functional characterization of *NRT2.3* is yet to be performed, and so the importance of this is currently unclear. The eighth putative nitrate transporter gene encodes CHLORIDE CHANNEL-A (*CLCA*). Not only was this gene found to mediate nitrate accumulation in plant vacuoles in *A. thaliana* (De Angeli et al., 2006), but it was also identified as a contributor to the control of leaf ${\text{NO}}_{3}^{-}$ homeostasis in a previous association analysis in *B. napus* (Koprivova et al., 2014). Thus, this is a very convincing candidate gene for controlling leaf ${\text{NO}}_{3}^{-}$ concentration. The gene encoding an ammonium transporter, specifically AMMONIUM TRANSPORTER 2 (*AM2*; Table 1), was identified on chromosome A4, close to a SNP at which locus allelic variation confers 2.1-fold variation in leaf ${\text{NO}}_{3}^{-}$ concentration (Figure 3). The encoded protein is thought to be localized to the plasma membrane, and experiments in *A. thaliana* indicate that it is a high-affinity transporter, expression of which is repressed by higher exogenous ammonium nitrate concentrations (Sohlenkamp et al., 2002). Whilst it seems clear that ${\text{NH}}_{4}^{+}$, and not ${\text{NO}}_{3}^{-}$ is the substrate for this protein, it is likely that changes in ${\text{NH}}_{4}^{+}$ uptake efficiency will impact on leaf ${\text{NO}}_{3}^{-}$ concentration and hence this association is perhaps unsurprising.

A further, highly interesting candidate for leaf ${\text{NO}}_{3}^{-}$ concentration is NIN (nodule inception)-LIKE PROTEIN 7 (*NLP7*). Allelic variation in the nearby lead-marker within the strong association peak on chromosome A1 confers 1.9-fold variation in leaf ${\text{NO}}_{3}^{-}$ concentration (Figure 3). *NLP7* has recently been shown to modulate expression of the major ${\text{NO}}_{3}^{-}$ sensor and transporter encoding gene *NRT1.1* (Zhao et al., 2018). As a ${\text{NO}}_{3}^{-}$ sensor, NRT1.1 appears to enable detection of ${\text{NO}}_{3}^{-}$-rich soil patches, leading to subsequent proliferation of roots in these areas (Bouguyon et al., 2016). This protein can also act as a dual-affinity transporter, depending on the phosphorylation status of a threonine residue within the protein, and is active at a range of exogenous ${\text{NO}}_{3}^{-}$ concentrations (Parker and Newstead, 2014). The association of SNP markers within LD decay of *NLP7* provides further evidence of its downstream role in ${\text{NO}}_{3}^{-}$ uptake in *B. napus*. This gene provides an excellent candidate for further study, allelic variants in which may provide a suitable route toward improvement of ${\text{NO}}_{3}^{-}$ uptake and N fertilizer use efficiency in *B. napus*.

The association of SNP markers close to the genes described here is indicative of variation in expression of these genes across the diversity population, which may subsequently impact on ${\text{NO}}_{3}^{-}$ uptake and leaf accumulation. Whilst the corresponding GEMs were not associated with the trait, it is important to state that the GEMs used in this study are based on transcript abundance in young leaves. Hence, genotype-specific differences in the expression of genes in roots or at different growth stages are likely to be masked. Similarly, variation in the expression of genes that are specifically expressed in root tissue, and not expressed in leaves, will not be reflected here. Thus, further work will be required to confirm differences in expression of such genes between genotypes in the RIPR diversity population.

### Orthologs of three ABC transporters required for root suberin synthesis likely contribute to nitrate and phosphorus homeostasis

Three and one gene(s) encoding putative ABC transporters were identified close to SNPs highly associated with leaf ${\text{NO}}_{3}^{-}$ and P concentration respectively (Table 1). These are orthologous to the three unique *A. thaliana* ABC-2 transporters G2 (AT2G37360), G6 (AT5G13580) and G20 (AT3G53510). All of these genes are expressed in roots and thought to be required for the transport of aliphatic polymer precursors of suberin or related structural components of the Casparian strip (Yadav et al., 2014). These genes also appear to be required for synthesis of an effective suberin barrier in seed coats, since *A. thaliana* plants mutated in these genes had increased seed permeability to tetrazolium red dye (Yadav et al., 2014). Suberin is considered to be a major component of the Casparian strip in roots which forms a chemical barrier restricting extracellular transport of water and dissolved nutrients to the vascular tissue (Baxter et al., 2009). This enables greater control of solute transport between plant tissues. *Arabidopsis thaliana* triple mutants in the aforementioned ABC transporter encoding genes were previously characterized and root systems were found to be more permeable to water as well as the solutes sodium chloride (NaCl) and potassium nitrate (KNO_3_; Yadav et al., 2014). The authors also deduced that whilst the mutant plants were able to make suberin, its structure was altered compared to wild-type plants. That all three of these genes were identified close to SNPs highly associated with leaf ${\text{NO}}_{3}^{-}$ and P concentration in this study provides further evidence for the role of suberin in the control of nutrient uptake in *B. napus*.

### Flowering time is an important marker for leaf nitrate and phosphorus concentration

Within LD decay of highly associated markers for leaf ${\text{NO}}_{3}^{-}$ and P concentration, were a number of genes thought to control flower development. These include orthologs of *A. thaliana* FLOWERING LOCUS C (*FLC*), a flowering time regulator, which were detected close to association peaks for leaf ${\text{NO}}_{3}^{-}$ and P concentration on chromosomes A3 and C9, respectively, as well as orthologs of PISTILLATA (*PI*), and AGAMOUS-LIKE 29 (*AGL29*) close to association peaks for leaf P concentration on chromosomes C2 and C4, respectively (Table 1). Allelic variation at the lead-marker close to *FLC* on chromosome A3 is associated with 1.9-fold variation in mean leaf ${\text{NO}}_{3}^{-}$ concentration (Figure 3). Allelic variation close to the *FLC* ortholog on chromosome C9 is also associated with 1.2-fold variation in leaf P concentration (Figure 4). Interestingly, orthologs of *FLC* and other flowering time regulating genes were previously found within LD decay of association peaks for leaf Ca and Mg concentration (Alcock et al., 2017). Expression of *FLC* directly influences flowering time by suppressing activation of flowering until after sufficient cold treatment (vernalisation; Sheldon et al., 2000). It has previously been demonstrated that winter cultivars of *B. napus*, which have higher vernalization requirements, have greater *FLC* transcript abundance than spring cultivars, which have lower vernalization requirements (Tadege et al., 2001). In the present study, leaf ${\text{NO}}_{3}^{-}$ concentration was found to vary between spring and winter crop types. Similarly, leaf P concentration data used for association analyses here was previously shown to vary between winter and spring crop types (Thomas et al., 2016). It is possible that the associations with flowering time regulator genes observed here and in previous studies are linked to spurious correlations between leaf nutrient concentration and flowering time between different crop types. However, it was previously shown that leaf N concentrations decline with leaf expansion during crop development (Liu et al., 2018). Hence, it is also possible that expression levels of *FLC* and other flowering time related genes could influence leaf nutrient concentrations indirectly by changing the duration of vegetative growth. Whilst further work will be required to elucidate the mechanisms controlling this association, the tendency of flowering time genes to associate with multiple nutrient traits indicates that they are suitable markers for leaf mineral concentration traits in *B. napus*.

### Root hair development genes are particularly important for leaf phosphorus accumulation

No SNPs or GEMs were significantly associated with leaf P concentration above the FDR corrected significance threshold in this study. This indicates that unlike leaf ${\text{NO}}_{3}^{-}$ concentration, leaf P concentration is under relatively low genetic control, or at the very least, that there are low levels of genetic variation that associate with variation in the trait. This is surprising, as variance components analysis in this population previously showed that 20% of the variation in leaf P concentration was associated with genotype, indicating a moderate genetic component (Thomas et al., 2016). However, this result is consistent with a previous association study in a similar population of *B. napus*, in which although heritability of shoot P concentration was estimated at 0.43, no significant marker associations were detected for the trait (Bus et al., 2014). It is possible that the markers used in both of these studies did not fully reflect genetic variation controlling shoot P accumulation. Despite this, a number of notable association peaks were detected in the Manhattan plot generated from SNP-based association data (Figure 2C). Allelic variation in lead-marker positions within these loci were also associated with variation in leaf P concentration (Figure 4), hence indicating that genes within LD of these markers are likely to contribute to control of the trait.

The three phosphate transporter encoding genes PHOSPHATE TRANSPORTER (*PHT*) 1.4, 1.7 and 3.1 were located close to highly associated SNP markers on chromosomes C4, A6, and C9, respectively. The former two genes appear to encode high-affinity transporters for external phosphate uptake (Shin et al., 2004; *PHT1.7* function inferred by sequence similarity), and thus their proximity to markers associated with leaf P concentration is unsurprising. The latter transporter is expressed in mitochondria, and appears to modulate plant salt stress responses (Zhu et al., 2012). Hence, whilst it remains an interesting candidate to consider, it is less likely than the former transporters to be the causative gene within its respective association peak. A further candidate which may have a direct influence on phosphate uptake is PHOSPHATE 2, otherwise known as UBIQUITIN-CONJUGATING ENZYME 24 (Huang et al., 2013). This gene has been shown to be involved in the response to exogenous phosphate concentration through degradation of PHOSPHATE TRANSPORTER 1 (PHO1) under phosphate sufficiency (Liu et al., 2012). PHO1 is crucial for phosphate loading to the xylem and subsequent transport to the shoot (Hamburger et al., 2002). Hence, alterations in expression or protein structure of PHOSPHATE 2 may provide a suitable avenue for improving P-fertilizer use efficiency, through altering the plant's response to exogenous phosphate supplies.

A number of candidate genes that may contribute indirectly to the accumulation of phosphorus were also identified, including genes within LD of SNPs highly associated with leaf P concentration on chromosome C8 that play a role in root system architecture. The genes, *EXPANSIN A7*, and *ROOT HAIR SPECIFIC 2* are both crucial for root hair elongation; *A. thaliana* mutants in these genes have shorter root hairs (Won et al., 2009; Lin et al., 2011). A further gene identified in close proximity, known as *KEULE*, plays a role in cytokinesis through proper vesicle tethering, which among other things is required for polar growth of root hairs (Wu et al., 2013). Root hairs have previously been linked to phosphorus acquisition, which increase in length and number under low exogenous supply or deficiency (Lynch, 2011; Wang et al., 2018). It has also recently been shown that the expression of auxin-inducible transcription factors is increased in root hairs and that auxin accumulates in root hair zones under low exogenous P conditions, hence promoting root hair elongation (Bhosale et al., 2018). It is clear that root hair traits can influence P accumulation by plants, and there is genetic variation in this trait that could be exploited to improve P acquisition and P fertilizer use efficiency (Wang et al., 2018).

Markers used for association analyses in this study were developed from leaf mRNA-sequence data. Whilst SNPs called from this data form suitable markers for identifying genetic differences between genotypes across all tissue types and developmental stages, GEMs based on leaf transcript abundance are only suitable for assessing genetic variation within young leaf tissue. Many of the candidate genes for leaf P concentration are predominantly involved in regulating root development and root specific transport. It is possible that stronger trait/marker associations would have been identified if more root-specific markers, such as root transcript abundance, had been utilized. Whilst significantly further work is required in order to generate sequence data required for calling such markers, this may be a suitable next step that could lead to the identification of many more molecular markers for nutrient acquisition in *B. napus* in the future.

### Allelic-variant analysis indicates scope for marker-assisted selection for improved nitrate and phosphorus use efficiency

Investigation into the effects of allelic variation at lead-marker positions for leaf ${\text{NO}}_{3}^{-}$ concentration indicated that huge differences in trait values between genotypes can be detected whilst only considering few, individual SNPs. For instance, a SNP in the lead-marker within an association peak on chromosome A4 was shown to confer over 2-fold mean variation in leaf ${\text{NO}}_{3}^{-}$ concentration between genotypes (Figure 3). Significant differences in leaf P concentration between allelic variants were also observed. However, the observed effects were of a lower magnitude than those observed for leaf ${\text{NO}}_{3}^{-}$ concentration. For instance, the greatest fold change between allelic variants was only 1.15, for lead-markers on chromosomes C2 and C9 (Figure 4). It is thus likely that there is still significant variation in the genetic basis of leaf P accumulation to be uncovered. This seems particularly convincing considering huge differences in P use efficiency previously identified within a diverse population of *B. oleracea* genotypes grown under varying exogenous P supply (Hammond et al., 2009) As discussed above, such variation could be realized by looking into root gene expression, as this data was not reflected by the leaf mRNA-sequence-based GEMs used in analyses here. It may also be worth quantifying P concentrations in specific cellular compartments. Under P sufficient conditions, up to 95% of intracellular P can be found in the vacuole (Yang et al., 2017). Whilst measuring vacuole-specific P concentrations across all 383 genotypes in the RIPR diversity population represents a difficult task, this could be a better measure of P status between genotypes, enabling identification of variation more closely associated with P use efficiency. Despite this, the identification molecular markers that associate with significant variation in leaf ${\text{NO}}_{3}^{-}$ and P concentration here may prove useful in marker-assisted selection strategies to improve nutrient accumulation or use efficiency in *B. napus*. This could accelerate the development of suitable crop varieties for a changing world, which is likely to demand greater yield from crop plants whilst reducing fertilizer inputs.

### Shoot gene expression is likely to play a role in the control of potassium homeostasis

As with leaf phosphorus concentration, no SNP markers were significantly associated with leaf K concentration in the RIPR diversity population. Similarly, no associations were identified for shoot K concentration in an association study in a different population of 509 *B. napus* genotypes (Bus et al., 2014). Thus, it is possible that leaf K concentration is under lower genetic control than leaf ${\text{NO}}_{3}^{-}$ concentration. However, there were several GEMs that had –log_10_*p*-values over the FDR corrected significant threshold. The GEM most highly associated with leaf K concentration is orthologous to *A. thaliana* At2g04850. Whilst relatively little is known about this gene, data from the SMART web-based tool (Schultz et al., 1998) indicates that it is membrane bound, and that it contains a Cytochrome b-561 / ferric reductase transmembrane domain. This gene is also thought to be auxin-responsive. Auxin is a plant hormone that is responsible for orchestrating multiple growth and development processes (Swarup and Bennett, 2003). Hence, it is possible that expression of this gene is related to development stage, which may correlate with leaf K concentration. Whilst the link between this gene and leaf K concentration is currently unclear, this was by far the most highly associated GEM, and hence is worthy of further consideration. A further GEM of interest underlies a gene orthologous to *A. thaliana, POTASSIUM TRANSPORTER 3* (*KT3*), also known as *TINY ROOT HAIR 1* (Daras et al., 2015). Whilst not associated with leaf K concentration above the FDR corrected significance threshold, the GEM corresponding to this gene is relatively highly associated with the trait, and shoot gene expression is positively correlated (Figure 5). Experiments in *A. thaliana* showed that KT3 knockout lines, which have significantly smaller root hairs, have a reduced rate of K transport, although not enough for the plants to develop deficiency symptoms (Rigas et al., 2001). The presence and length of root hairs have also been correlated with increased K acquisition among plant species (White, 2013; Hinsinger et al., 2017). Thus, genetic variation in this trait might be exploited to improve K acquisition and K fertilizer use efficiency. It is perhaps surprising that further known K^+^ transporters were not identified by association analyses performed here. However, this may be a product of using leaf mRNA-sequence data to develop GEMs. As discussed above with respect to leaf P concentration candidates, such markers do not reflect root gene expression differences between genotypes. Within LD decay of co-localizing GEM association peaks on chromosomes A9 and C9 are also genes orthologous to *A. thaliana* AT1G05940.1, a gene that encodes a cationic amino acid transporter. This may play a role in K acquisition within *B. napus*. However, association peaks in the same loci were detected for seed glucosinolate content (Lu et al., 2014). Over approximately the last 50 years, intensive breeding programmes have been undertaken to reduce seed glucosinolate concentrations in *B. napus* (Allender and King, 2010). Due to such strong selection pressures, it is possible that a number of other traits were perturbed in the process, and hence the association peaks on chromosomes A9 and C9 detected here may be a result of unconscious co-selection.

It is clear from SNP and GEM association analyses that transcript abundance forms a better marker for elucidating genes controlling leaf K concentration than sequence polymorphisms between genotypes. This also consistent with results from a previous study in *B. napus* in which no SNPs were associated with leaf K concentration (Bus et al., 2014) and in spinach in which only two SNPs associated with leaf K concentration, whereas a greater number of associations were observed for other minerals (Qin et al., 2017). To our knowledge, this is the first time that leaf transcript abundance has been used as a molecular marker to identify loci controlling leaf K accumulation. However, correlations between transcript abundance of candidate genes and leaf K concentration were relatively low (*r*^2^ < 0.1; Figure 5). Whilst the use of such genes in marker assisted selection strategies may be possible, the identification of genes that have a greater influence over trait control is desirable. It is possible that examining the effects of root gene expression across the population would lead to the discovery of further such genes. Root traits have previously been correlated with plant K acquisition (White, 2013; Hinsinger et al., 2017), and genotype-specific gene expression within this tissue is likely to reflect variation in K uptake and consequently leaf K concentration. However, root gene expression data across a diversity population of *B. napus* is not available at this time. Despite the limitations of using shoot gene expression data for the determination of genes controlling leaf K concentration, a number of convincing candidate genes have been identified, expression of which could be modified to alter K accumulation and use efficiency.

## Conclusions and perspective

This study has identified large amounts of variation in leaf ${\text{NO}}_{3}^{-}$ concentrations across 383 genotypes of *B. napus* grown in controlled conditions. This likely reflects the majority of species-wide heterogeneity in the trait and demonstrates the scope for breeding for increased ${\text{NO}}_{3}^{-}$ use efficiency through traditional techniques. This study has also determined genetic loci and allelic variants associated with leaf ${\text{NO}}_{3}^{-}$, P and K concentrations in *B. napus*. Several convincing candidate genes that may affect these traits either directly or indirectly have also been identified. These include genes which relate to suberin synthesis and root hair development, both of which are likely to be amenable traits for improving uptake and use efficiency of multiple nutrients (White et al., 2013). Characterizing variation in traits related to suberin synthesis and root hair development across genotypes of the RIPR diversity population would likely add to the understanding of their function and how they can be applied in crop breeding strategies. Whilst numerous candidate genes for leaf accumulation of ${\text{NO}}_{3}^{-}$, P and K have been discussed here, each of these were largely identified using functional data available for *Arabidopsis thaliana* orthologous genes. It is likely that there are many other genes associated with the traits measured here that are yet to be identified, including genes within association peaks in which no candidate genes were identified. Some of these could represent novel targets for the control of nutrient uptake and distribution in *B. napus*, functions of which are yet to be revealed. Further investigation into Associative Transcriptomics data produced here may help to elucidate these in the near future. Allelic variants associated with variation in leaf ${\text{NO}}_{3}^{-}$, P and K concentration identified here also provide an excellent resource for marker-assisted selection. Together, these results make significant progress in the understanding of nutrient homeostasis in *B. napus*, which may prove useful in the generation of more nutrient and fertilizer use efficient crop varieties.

## Data availability

Transcriptome sequences used in Associative Transcriptomics analyses are deposited within the Sequence Read Archive (Leinonen et al., 2011) under accession number PRJNA309367. Raw, Associative Transcriptomics outputs supporting the conclusions of this manuscript will be made available by the authors, without undue reservation, to any qualified researcher.

## Author contributions

MB, IB, PW, TA, and NG conceived the project and contributed to experimental design. TA analyzed leaf nitrate and Associative Transcriptomics data. LH and ZH prepared functional genotypes and performed Associative Transcriptomics. LW prepared leaf samples and carried out ion chromatography analyses. TA and NG wrote the manuscript. All authors contributed to and have read and approved the final version of the manuscript.

### Conflict of interest statement

The authors declare that the research was conducted in the absence of any commercial or financial relationships that could be construed as a potential conflict of interest.
