# Supplementary figures and images for: Species-Wide Variation in Shoot Nitrate Concentration, and Genetic Loci Controlling Nitrate, Phosphorus and Potassium Accumulation in Brassica napus L
Source: Front Plant Sci. 2018 Oct 16;9:1487. doi: 10.3389/fpls.2018.01487 (PMC6198146; doi:10.3389/fpls.2018.01487)

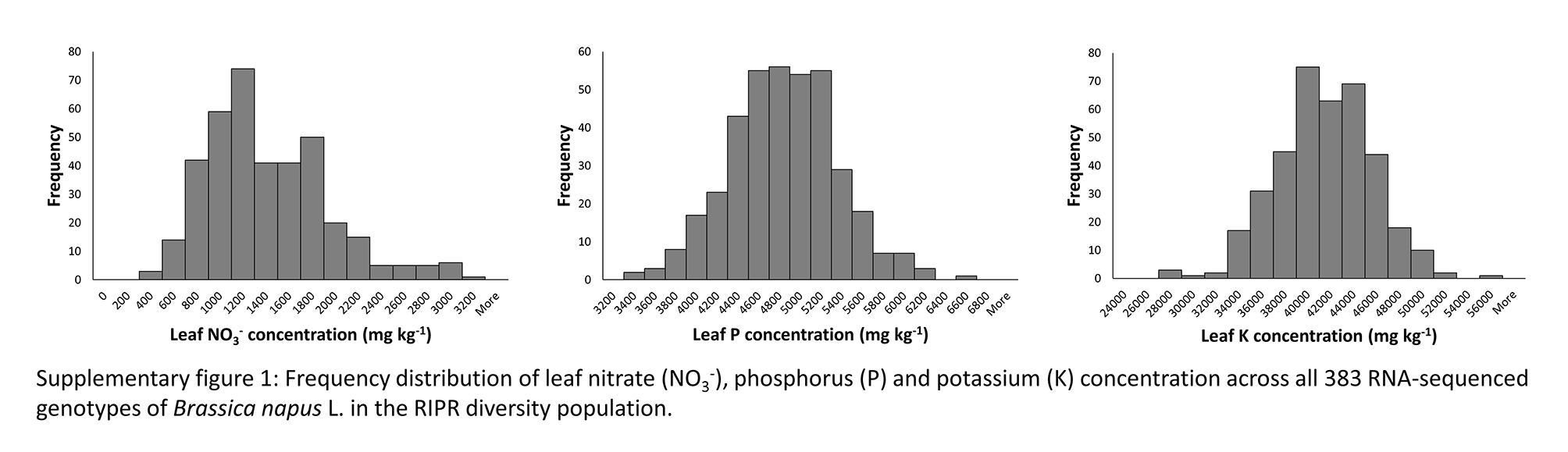

Supplement: Supplementary file 1 [file Image_1.TIF]

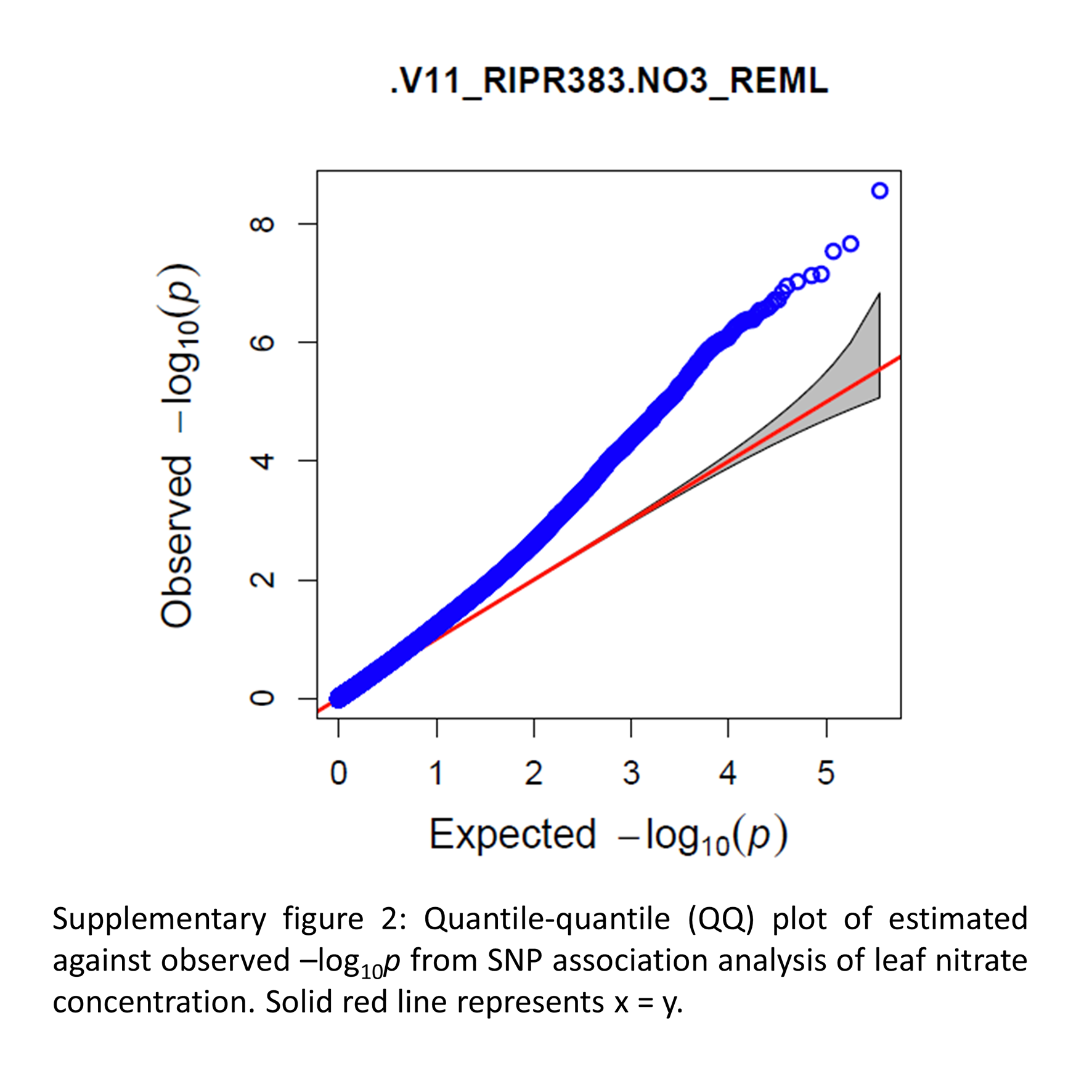

Supplement: Supplementary file 2 [file Image_2.TIF]

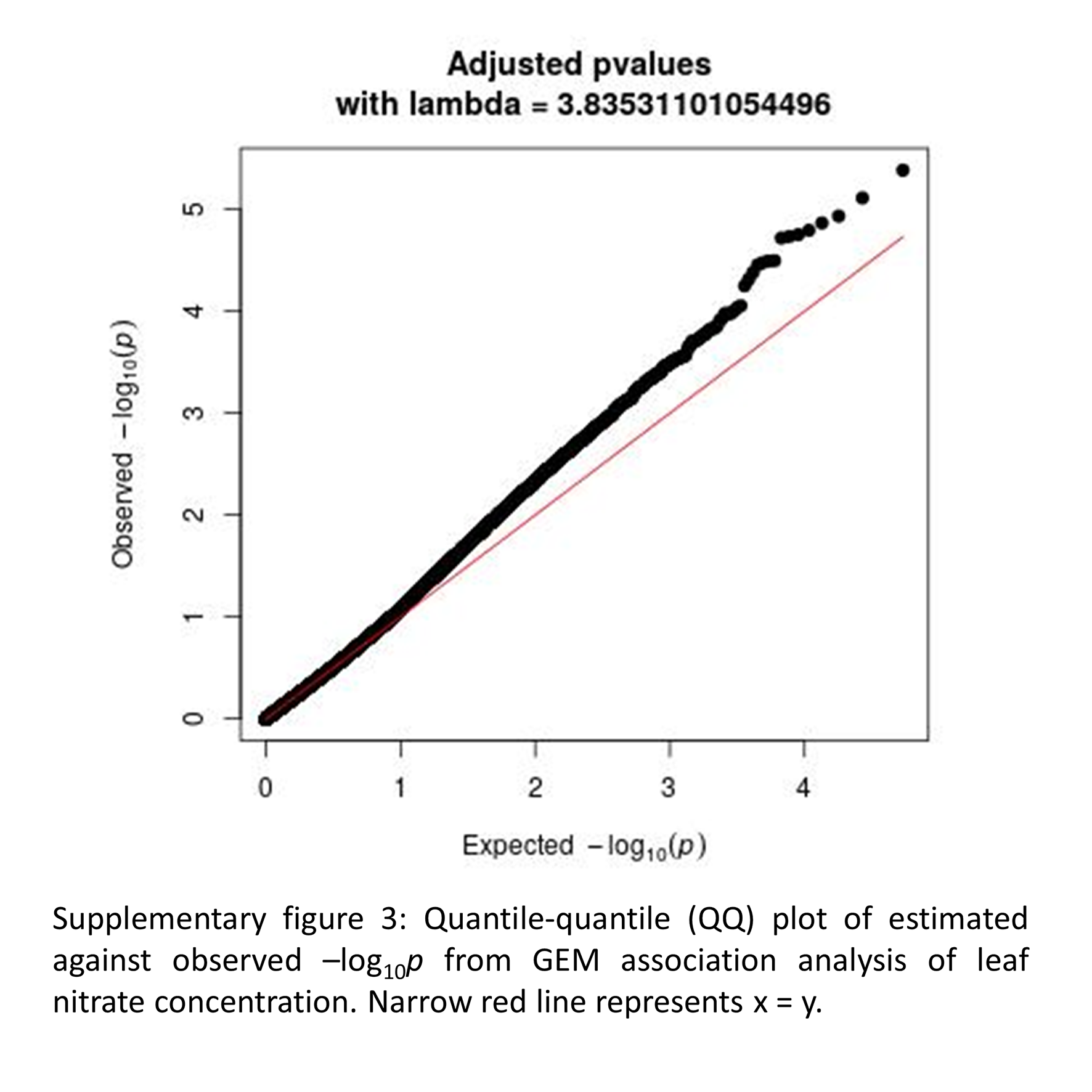

Supplement: Supplementary file 3 [file Image_3.TIF]

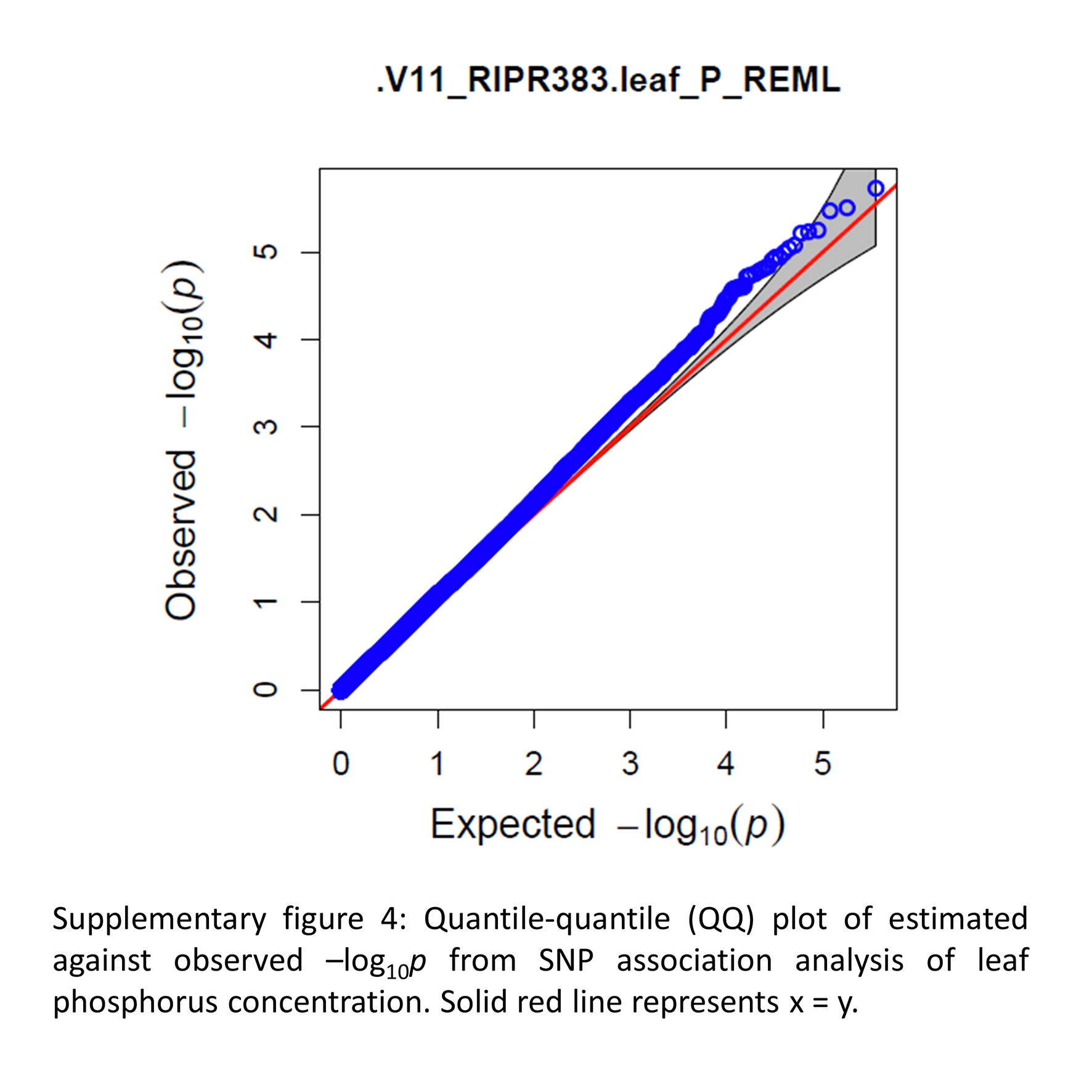

Supplement: Supplementary file 4 [file Image_4.TIF]

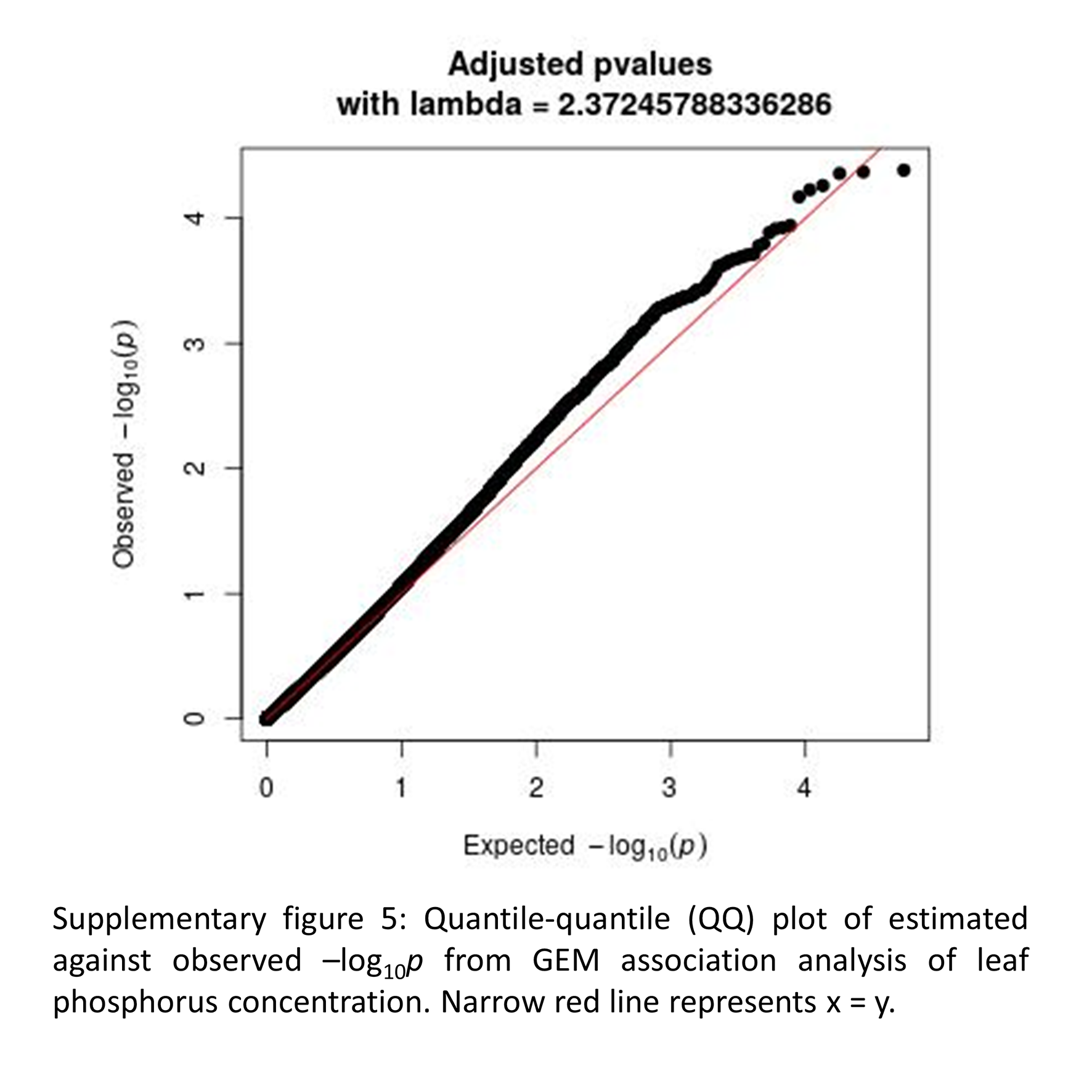

Supplement: Supplementary file 5 [file Image_5.TIF]

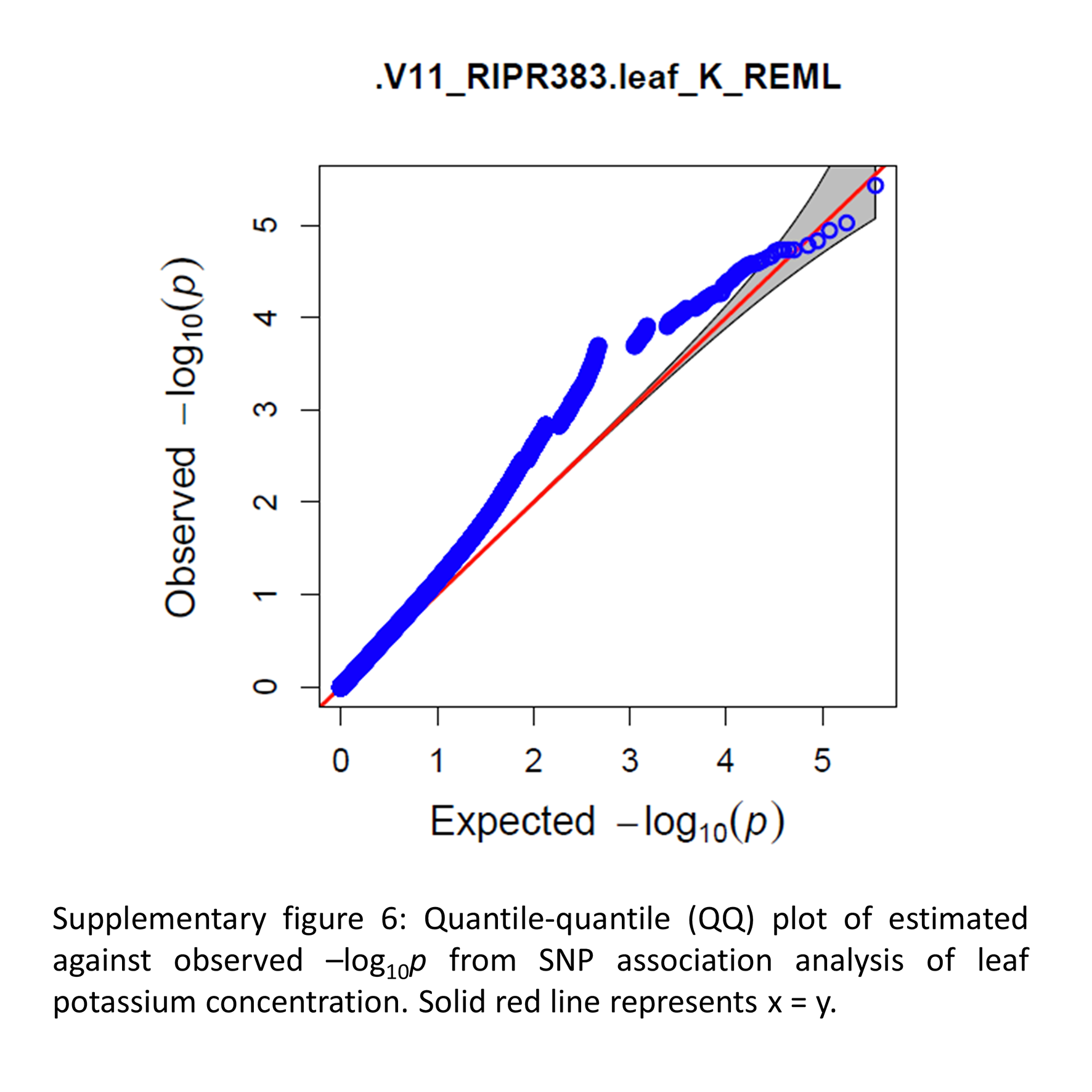

Supplement: Supplementary file 6 [file Image_6.TIF]

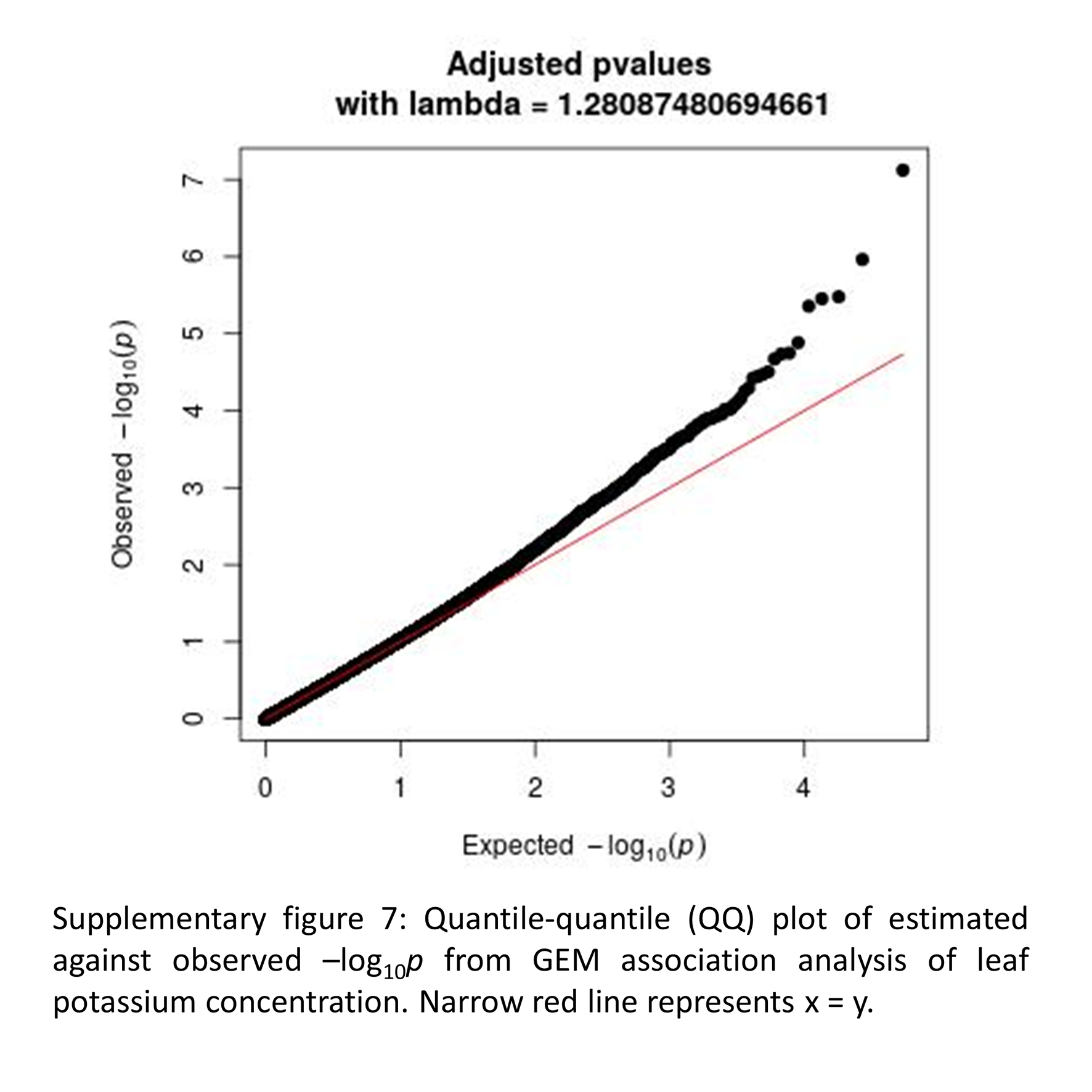

Supplement: Supplementary file 7 [file Image_7.TIF]
